# An examination of sustained attention during complex multitasking scenarios

**DOI:** 10.1186/s41235-025-00674-x

**Published:** 2025-10-07

**Authors:** Jonathan C. Rann, Amit Almor

**Affiliations:** 1 Department of Psychology, University of South Carolina, Columbia, SC 29208 USA; 2 Institute for Mind and Brain, University of South Carolina, Columbia, SC 29208 USA; 3 Linguistics Program, University of South Carolina, Columbia, SC 29208 USA

**Keywords:** Sustained attention, Vigilance decrements, Multitasking

## Abstract

We report results from two experiments that examined the time course of vigilance decrements during a demanding multitasking scenario. Specifically, we implemented a novel paradigm in two experiments in which a total of 123 participants performed a go-no-go target detection continuous performance test (CPT) task simultaneously with a driving-based tracking task. Growth curve analyses of the temporal trajectories of performance of both tasks revealed vigilance decrement effects that varied across CPT and tracking measures, and between different target presentation rate conditions. Our findings highlight the importance of executive function, arousal, and motivation in such dual-task performance and support a multifaceted approach combining elements from the cognitive overload, cognitive underload, and opportunity-cost models of vigilance decrements. Insights from this work can inform the design and development of complex operator–system interfaces and thus increase safety and effectiveness for operators during mission-critical situations.

## Significance statement

Many real-world occupations, in both the civilian and military worlds, require operators to maintain adequate levels of alertness to detect and respond to critical signals over long periods. However, this can be difficult due to the well-documented vigilance decrements, which are reflected in the gradual deterioration of several aspects of performance. For example, in tasks that require responding to some events (targets) while avoiding others (non-targets), target hits typically decrease and reaction time typically increases as operators spend more time on the task. Researchers often rely on target detection tasks known as continuous performance tests (CPTs) to investigate the source of these decrements in highly controlled laboratory settings. However, these powerful tools are seldom used to measure performance during demanding real-world multitasking scenarios. In this article, we report the results from two experiments in which participants simultaneously performed a driving-based target tracking task with a go-no-go target detection CPT task for approximately 12 min. Results revealed changes in the onset of vigilance decrements across measures, especially when CPT target presentation rates were faster than when they were slower. Together, these findings support the cognitive overload, cognitive underload, and opportunity-cost models of vigilance decrements and demonstrate that executive function, arousal, and motivation are important psychological constructs involved in sustained attention and multitasking performance. Insights from this work can inform the design and development of complex operator–system interfaces and thus increase safety and effectiveness for operators during mission-critical situations.

## Introduction

Sustained attention, generally defined as the ability to continuously engage in relevant activities (Esterman & Rothlein, 2019; Fortenbaugh et al., 2017; Sarter et al., 2001), is a fundamental aspect of many tasks that require operators to detect and respond to critical signals over long periods (e.g., military surveillance, drone operation, long-distance truck driving) (Cardoso et al., 2019; Dorrian et al., 2007; Ghylin et al., 2007; Hancock & Hart, 2002; Heikoop et al., 2017; Künzell et al., 2018; Masoudian & Razavi, 2019; Reinerman-Jones et al., 2016). While performing such tasks, operators must exert varying levels of control for meeting task requirements while also maintaining adequate arousal in order to handle other, unexpected events (Ballard, 1996; Fernandez-Duque & Posner, 2001; Hancock, 1989; Klösch et al., 2022; Langner & Eickhoff, 2013; van Schie et al., 2021). This has been shown to be difficult, as vigilance decrements typically occur in which several measures of performance, such as target hit rate (hits) and reaction time (RT), deteriorate as time-on-task increases (Dillard et al., 2019; Mackworth, 1948; Parasuraman et al., 1987).

Much of the research on vigilance decrements has relied on sustained attention paradigms to investigate their underlying mechanisms under highly controlled experimental settings (for review, Riccio et al., 2002). These tools allow researchers to systematically explore how specific task features can influence operator performance during demanding contexts (Fisk & Scneider, 1981; Parasuraman & Mouloua, 1987; Roca et al., 2011), and how systematically modulating arousal can shape these effects (Buckley et al., 2016; Conners, 2014; He & McCarley, 2011; Luna et al., 2021a, 2021b, 2022a, 2022b; McBride et al., 2007). Understanding vigilance decrements is particularly important for real-life scenarios in which operators perform multiple simultaneous tasks as a function of their job requirements (Chen & Joyner, 2009; Chérif et al., 2018; Karpinsky et al., 2018; St. John et al., 1997; Strayer et al., 2003, 2011, 2019; Tillman et al., 2017). However, few studies have used sustained attention paradigms to closely examine multitasking performance in settings that simulate the complexity of real-world operations. To address this gap, the present study integrates a sustained attention task that yields multiple performance measures with a continuous driving-based tracking task to examine vigilance decrements in a controlled dual-task scenario that mirrors real-world demands.

### Sustained attention and vigilance

Sustained attention is supported by the dynamic coordination of the multiple cognitive systems involved in goal-directed information processing (Fortenbaugh et al., 2017; Mirsky et al., 1991; Posner & Petersen, 1990; Sarter et al., 2001). Key among these are selective attention, which involves focusing cognitive resources on a particular stimulus or task goal (Johnston & Dark, 1986; Kahneman, 1973; Navon & Gopher, 1979; Treisman, 1969), and divided attention, which requires managing resources across multiple stimuli or task goals (Allport et al., 1972; Black et al., 2022; Janczyk & Kunde, 2020; Koch et al., 2018; Poljac et al., 2018; Wickens, 2008). These systems are further supported by higher-order mechanisms, most notably working memory and executive function, which store, maintain, and coordinate the processing of task-relevant information (Allport et al., 1972; Baddeley & Hitch, 1994; Black et al., 2022; Lara et al., 2014; Poole & Kane, 2009; Kieras et al., 2000; Kieras & Meyer, 1997; Koch et al., 2018; Poljac et al., 2018; Rubinstein et al., 2001; Sternberg, 1969; Wickens, 2002).

Although sustained attention is often treated as a distinct construct, it nonetheless overlaps conceptually and functionally with the notion of *vigilance* (Head, 1923; Klösch et al., 2022; MacLean et al., 2009; Oken et al., 2006; van Schie et al., 2021). Vigilance has been described as a state of readiness to detect subtle or infrequent changes in the environment (Mackworth, 1968) and involves physiological components such as alertness, defined as a sensitivity to incoming stimuli, and arousal, defined as the overall degree of alertness across states like sleep and wakefulness (Posner, 2008). In comparison with sustained attention, which reflects focused, goal-directed readiness for specific stimuli or tasks, vigilance encompasses a broader, more diffuse readiness to detect any relevant environmental change (van Schie et al., 2021). In this sense, maintaining vigilance may thus be necessary for sustained attention, but the opposite is not true. While these distinctions are conceptually meaningful, the current work does not aim to disentangle them. Rather, we focus on how performance changes over time—particularly in relation to performance declines observed in real-world settings.

### Vigilance decrements

Research consistently shows that performance on sustained attention tasks declines over time (e.g., Dember et al., 1992; Dillard et al., 2019; Ditchburn, 1943; Scerbo, 1998; Teichner, 1974; Warm et al., 2018; Wyatt & Langdon, 1932). These performance declines, commonly referred to as ‘vigilance decrements,’ were first systematically studied by Norman Mackworth during World War II, when he observed declining detection accuracy in radar operators over prolonged periods of monitoring (Baca, 2017; Lichstein et al., 2000; Mackworth, 1948). Building on Mackworth’s foundational work, subsequent research investigated the task-specific factors that influence the rate and severity of these performance declines (Lanzetta et al., 1987; Parasuraman, 1979). Notably, Parasuraman and Davies (1977) developed a ‘vigilance taxonomy’ that links task characteristics to performance outcomes and posits that the likelihood, onset speed, and magnitude of vigilance decrements are shaped by task demands (Hancock et al., 2016; Matthews & Davies, 1988; Robinson & Brewer, 2023; See et al., 1995; Unsworth & Robison, 2020).

These insights have given rise to several theoretical accounts that attempt to explain the underlying mechanisms of vigilance decrements. These accounts are commonly classified into two main types. *Resource depletion* (or cognitive overload) theories argue that sustained performance tasks are stressful and demanding and that decrements are related to progressive declines in working memory capacity as time-on-task increases (Caggiano & Parasuraman, 2004; Fisk & Scerbo, 1987; Gartenberg et al., 2018; Helton & Warm, 2008; Matthews et al., 1993; Wiener et al., 1984). In contrast, *cognitive underload* theories emphasize that sustained attention tasks are monotonous and boring (Cummings et al., 2016; Greenlee et al., 2018; McBain, 1970; Scerbo et al., 1992) and that decrements are related to progressive declines in arousal leading to withdrawal of attention from the primary task. A third perspective, the *opportunity-cost account* (Kurzban et al., 2013), proposes that vigilance decrements occur when individuals reallocate cognitive resources based on cost–benefit tradeoffs, adjusting their response strategies to the perceived utility of continuing to sustain attention (Gyles et al., 2023; Thomson et al., 2016).

Together, these theories underscore the multifaceted nature of vigilance decrements, indicating that performance declines may stem from a complex interplay of cognitive demands, arousal fluctuations, and motivational considerations. While these accounts offer distinct explanatory frameworks, they are not mutually exclusive; rather, multiple mechanisms may operate in parallel within a given task context. This overlap often makes it difficult to isolate specific contributing factors in real-world scenarios, where different mechanisms can produce similar behavioral outcomes. Task context remains critical for interpretation, particularly in distinguishing between cognitive overload and underload, whereas opportunity-cost effects are often most apparent in dual-task settings in which performance deteriorates unevenly across tasks as attention is reallocated based on perceived utility.

### Challenges of measuring sustained attention

Although theoretical models offer valuable explanations for vigilance decrements, accurately measuring sustained attention remains a major challenge. To address this, researchers often rely on continuous performance tests (CPTs), which follow a blocked successive discrimination task paradigm in which participants are required to either respond, or withhold response, when detecting target stimuli among distractors (Ord et al., 2021; Riccio et al., 2002; Robertson et al., 1997; Rosvold et al., 1956; Smid et al., 2006). CPTs yield multiple performance metrics, including reaction time (RT), omission and commission errors, and detection accuracy (e.g., hits and correct rejections or CRs) (Borgaro et al., 2003; Edwards et al., 2007; Riccio et al., 2001; Roebuck et al., 2016). CPTs also support signal detection theory (SDT) analyses, including sensitivity (*d*′), which reflects the ability to discriminate between targets among non-targets, and response bias (*β*), which reflects a preference for speed (liberal bias) or accuracy (conservative bias) when responding to targets (Azizi et al., 2018; Green & Swets, 1966).

To extract meaningful insights from CPT data, these performance metrics are often averaged across the entire task to assess overall attention and analyzed across successive trial blocks to capture temporal dynamics in sustained attention (Bubnik et al., 2015; Cornblatt et al., 1989; Esterman & Rothlein, 2019). However, even averaging performance within a block can obscure important within-block fluctuations (discussed later), especially when target frequency or task pacing change during the block. Moreover, even knowing that accuracy changes within a block does not reveal *why* it changes, or which mechanisms (e.g., cognitive overload or waning arousal) drive the change.

To address limitations of block-wise averaging, the Conners’ Continuous Performance Test (CCPT; Conners, 2014) alternates short and long interstimulus intervals (ISIs) within blocks throughout the task. This design allows for disentangling different reasons for performance declines, as shorter ISIs likely impose higher demands on executive function and working memory, whereas longer ISIs likely impose higher demands on maintaining alertness over time (Conners et al., 2003; Egeland & Kovalik-Gran, 2010). By comparing performance declines between short and long ISIs over time (i.e., across successive blocks), researchers can pinpoint the likely reasons for these declines.

Beyond task design, additional challenges arise in how sustained attention is statistically modeled. Many studies rely on linear analysis across discrete time points (e.g., ANOVA, regression), which, as briefly mentioned earlier, can obscure important non-linear changes in performance trends (Winter & Wieling, 2016). These limitations are especially critical in applied settings where extended task exposure may lead to learning-based practice effects that can mask, delay, or even counteract the occurrence of vigilance decrements during the early stages of task engagement, particularly in tasks demanding higher-order executive function (Brown, 2008; Fisk & Schneider, 1981; Norman & Shallice, 1986).

**Fig. 1:**
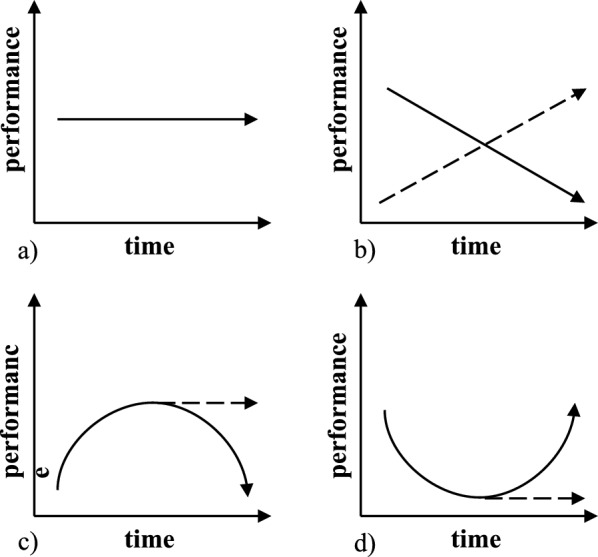
Schematic graph of possible performance trajectories. **a** Flat performance; **b** Linear performance indicating either progressive vigilance decrements (solid line) or practice effects (dashed line); **c**, **d** Curvilinear performance indicating (**c**) early practice effects and then later vigilance decrements (solid line) or flat performance (dashed line), or (**d**) early vigilance decrements and later practice effects (solid line), or flat performance (dashed line). Note that ‘better’ performance according to a hypothetical measure is illustrated by higher (more positive) values. In reality, in different CPT measures, better performance can be indicated by either higher (e.g., target hits) or lower (e.g., RTs and Omissions) values

Figure 1 illustrates how practice and vigilance decrement effects can shape performance trajectories. In minimally demanding tasks, performance may remain stable across blocks (Fig. 1a). In contrast, demanding multitasking scenarios can produce linear trends marked by either progressive declines due to vigilance decrements (Fig. 1b—solid line) or improvements due to practice effects (Fig. 1b—dashed line). Performance may also be characterized by curvilinear patterns, which progressively improve in the first few blocks due to early practice effects and then either degrade toward the end of the session due to late-onset vigilance decrements (Fig. 1c—solid line) or flatten due to task attenuation (Fig. 1c—dashed line). Alternatively, demanding multitasking scenarios can lead to opposite patterns in which performance progressively degrades in the first few blocks due to overloading executive resources by having to repeatedly task-switch. Performance can then either improve toward the last few blocks of the session due to late-onset practice effects (Fig. 1d—solid line), or flatten due to task attenuation (Fig. 1d—dashed line).

Given the theoretical accounts of vigilance decrements that we discussed, we can anticipate performance patterns aligned with those illustrated in Fig. 1. As noted earlier, disentangling overload from underload is notoriously difficult because both can produce performance declines over time. However, overload is more likely to emerge during short ISIs, whereas underload is more likely to emerge when stimulation is lower during long ISI blocks. Specifically, cognitive overload models predict steady linear declines (Fig. 1b—solid line) or late curvilinear declines (Fig. 1c—solid) when task demands are high (e.g., for short ISIs). In contrast, cognitive underload accounts predict the same patterns when task demands are low (e.g., for long ISIs). Finally, opportunity-cost accounts predict *divergent* trends such that improvements in one task may be coupled with declines in the other, or shifts in response bias as attention is redeployed to maximize utility.

In summary, sustained attention paradigms such as CPTs have been instrumental in advancing our understanding of vigilance decrements under controlled conditions. These tools have supported key theoretical accounts, including the cognitive overload and underload models. However, most prior work has focused on single-task environments and has not examined how these mechanisms unfold in more ecologically valid, multitasking scenarios that require continuous and concurrent engagement. Moreover, little attention has been given to how nonlinear performance trajectories may reveal different sources of decline. As a result, traditional-CPT findings may not fully generalize to real-world operational settings that demand dynamic coordination across tasks over extended periods (Chaytor & Schmitter-Edgecombe, 2003; Deniaud et al., 2015; Faria et al., 2023; Halperin et al., 1991; Sayer, 2005; Stojmenova & Sodnik, 2018; Thorpe et al., 2019).

### The present study

In the present study, we aimed to: (1) develop a clear method for systematically assessing performance changes across time in two simultaneous tasks that resemble many real-world scenarios and (2) evaluate whether the observed patterns are more consistent with cognitive overload, cognitive underload, opportunity costs, or a combination of these. To achieve these aims, we conducted two experiments which implemented a novel dual-task paradigm that we developed that integrated a go-no-go target detection task (Conners, 2014) with a smooth pursuit tracking task adapted from the continuous tracking and reaction (ConTRe; Mahr et al., 2012) paradigm in the OpenDS driving simulator environment (Math et al., 2013). We also analyzed time-related changes in performance using growth curve modeling (Baayen et al., 2008; Bates et al., 2014).

This dual-task framework offers a realistic testbed for understanding how sustained attention is managed in settings that require ongoing monitoring and rapid decision making, conditions that closely reflect many real-world scenarios (Abich IV et al., 2017; Chérif et al., 2018; Lee & Taatgen, 2019).

## Experiment 1

Our first question for this study was whether and how performance would change over time for each of the measures and indices associated with the CPT and tracking tasks. Previous studies have demonstrated that performance trajectories may vary in both shape and direction over time due to different factors, such as learning-based practice effects (Alexander et al., 2003; Beglinger et al., 2005) and vigilance decrements (Haubert et al., 2018; Lee et al., 2010; Pattyn et al., 2008). However, no previous study has specifically examined how these temporal trajectories unfold in a combined task paradigm, where a discrete CPT is performed alongside a continuous tracking task in an arrangement that introduces competing cognitive demands and may influence how vigilance-related changes manifest and are measured over time.

Our second question for this study was whether the temporal trajectories of any of our measures or indices mentioned above would differ when target presentation rates are faster compared to when they are slower. The Conners CPT (Conners et al., 2014) represents a well-established method in vigilance research, wherein interstimulus intervals (ISIs) are varied within task blocks to modulate attentional demands (Basner & Dinges, 2011; Conners et al., 2003; Egeland & Kovalik-Gran, 2010; MacLean et al., 2009). This design is based on the premise that longer ISIs place greater demands on sustained readiness to respond, thereby increasing the likelihood of detecting attentional lapses and enabling a more fine-grained assessment of performance over time. Despite its widespread use, this approach has not been applied to examine time-based performance patterns in dual-task settings where a CPT is paired with a continuous tracking task.

### Methods

#### Participants

Following the approval of the University of South Carolina IRB board, a total of 63 native English-speaking participants (age: *M* = 20.19, *SD* = 1.48) from the University of South Carolina Department of Psychology undergraduate participant pool took part in the experiment. Participants were compensated with extra credit for their time. We conducted a power analysis prior to testing in R (R Core Team, 2012) using the wp.rmanova function from the WebPower library (Zhang et al., 2018) for a medium effect-size (*η*^2^) =.50, alpha (*α*) =.05, and power (*β*) =.80. The analysis indicated a sample size of 58 participants, which we exceeded by 5 participants to allow for potential data loss. Of the 63 participants, 45 were female (age: *M* = 20.2, *SD* = 1.32) and 18 were male (age: *M* = 20.2, *SD* = 1.86). Recruitment criteria for this study specified that participants had to be native speakers of English and must have also held a valid driver’s license. There were no other inclusion or exclusion criteria for selecting participants.

#### Design

The design of this study was influenced by previous studies that examined task performance using combined multitasking paradigms (Buckley et al., 2016; Castro et al., 2019; Luna et al., 2022a, 2022b; Rann & Almor, 2022). Unlike studies that compared single- and dual-task performance using counterbalanced conditions (e.g., Chiew & Brazer, 2013; Moran et al., 2020), our primary aim was to examine within-session changes during dual-tasking, where the need for switching between the tasks may either exacerbate or mitigate vigilance decrements. To support this focus, we utilized a fixed-order design in which participants completed a single-task CPT session first, followed by a dual-task session. This approach ensured that participants were well-practiced in the demanding CPT so as to increase power for detecting vigilance decrements in both tasks during the second, dual-task, session (Carter et al., 2013; Cornblatt et al., 1989; Hope et al., 1998; Lemay et al., 2004).

Accordingly, we report the results for the two sessions separately. While we present the analyses from both sessions, we caution that we cannot derive any strong conclusion from performance differences between the sessions as the source of these differences can be either the order of the tasks, or the presence vs. absence of the secondary tracking task. Nevertheless, we note that measures that show similar result patterns in both sessions are likely not sensitive to either order or dual- vs. single-task differences.

#### Tasks

**Fig. 2:**
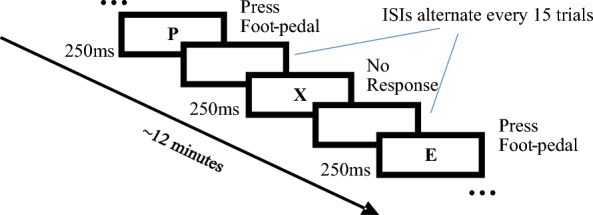
The CPT task. Participants are required to press the foot pedal when they detect a target letter and withhold response when a non-target letter is detected. The blank screen between stimuli lasted for either 1000 ms (short ISI), 2000 ms (middle ISI), or 4000 ms (long ISI)

The primary task was a go-no-go target detection task that was based on the Conner’s (2014) CPT and was coded and implemented using E-prime 3.0 stimulus presentation software (Psychology Software Tools, Pittsburgh, PA). It required participants to continuously monitor a computer screen as black letter stimuli (*font*: Arial; *size*: 200px) were presented against a white background, and to respond by pressing a foot pedal when they detected target letters (‘A’–‘W’, ‘Y’, ‘Z’) and withhold response when they detected the critical non-target letter ‘X’ (Fig. 2). The foot pedal was a Linemaster T-91-S Treadlite II Foot Switch attached to a Psychology Software Tools 200 A Serial Response Box that interfaced with the computer that ran the CPT.

Each stimulus presentation (whether target or non-target), as well as the subsequent time window including the ISI and up until the next stimulus presentation, was considered a trial. Each session consisted of 270 trials presented over the course of approximately 12 min with trials appearing at the predetermined rate regardless of whether the participant responded. Each session was divided into six blocks, each including 45 trials. Each block consisted of three sub-block conditions, each with a different ISI (1000 ms, 2000 ms, or 4000 ms). The order of the three sub-blocks was randomly determined for each block for each participant. Target and non-target stimuli were randomly selected with an 80–20% ratio of targets to non-targets, each presented for 250 ms. Performance data for this task was sampled per trial and stored in E-prime output data files. The primary CPT task was administered as the only task in the first session and then together with the secondary driving task in the second session.

**Fig. 3:**
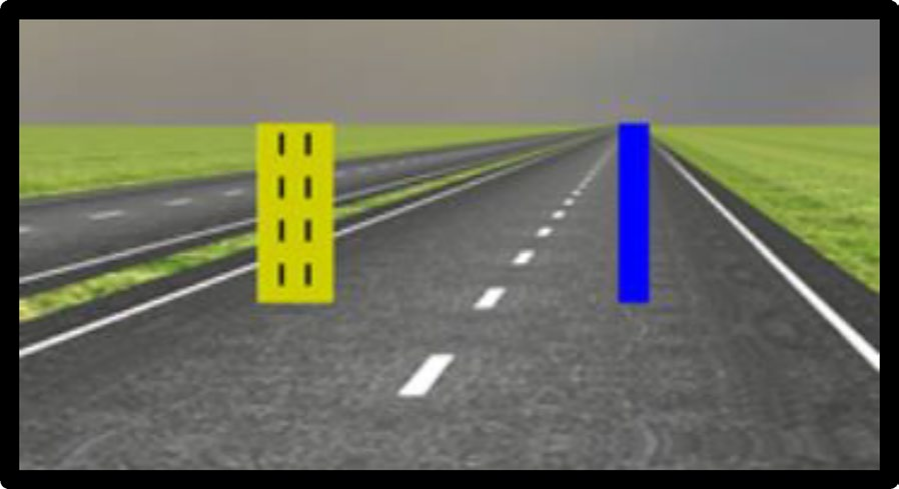
ConTRe task. Participants are required to track the moving yellow target using the blue cursor they control using a steering wheel

The secondary driving-based tracking task was the continuous tracking and reaction (ConTRe) task (Mahr et al., 2012) implemented in the OpenDS driving simulator (Math et al., 2013). It required participants to continuously track a yellow target moving across the simulator screen with a blue cursor that they controlled using a steering wheel (a Microsoft SideWinder Precision Racing Wheel) which interfaced with the computer that ran the simulator (Fig. 3).

The yellow target was placed approximately 20 simulated ft in front of the participants’ view on the simulator screen and moved horizontally (i.e., left-to-right, right-to-left) across the screen at a constant lateral speed of 1 simulated meter per second. The yellow target’s direction of movement (left-to-right, right-to-left) changed at random times. Participants only had control of the lateral movement of the blue cursor. Performance data for the tracking task was sampled approximately every 19 ms and stored in a MySQL database.

To allow for accurate temporal synchronization of the tracking and the CPT data, OpenDS was programmed to emit a sound that started the e-Prime CPT task in the second experiment session through a voice key.

#### Apparatus

The CPT ran on a Dell Precision Tower 7810 computer and was displayed on a Dell 17-inch monitor at a 1280 × 1040px resolution and a refresh rate of 60 Hz. The driving-based tracking task ran on a Dell XP 435t/9000 computer and was displayed on a Dell 27″ full HD 1920 × 1080 flat-panel monitor with a refresh rate of 60 Hz. This tracking task computer was used to collect data for approximately half the participants in E1 and was replaced by a Dell OptiPlex 790 computer after malfunctioning. Both computers ran the Windows 10 Pro operating system.

**Fig. 4:**
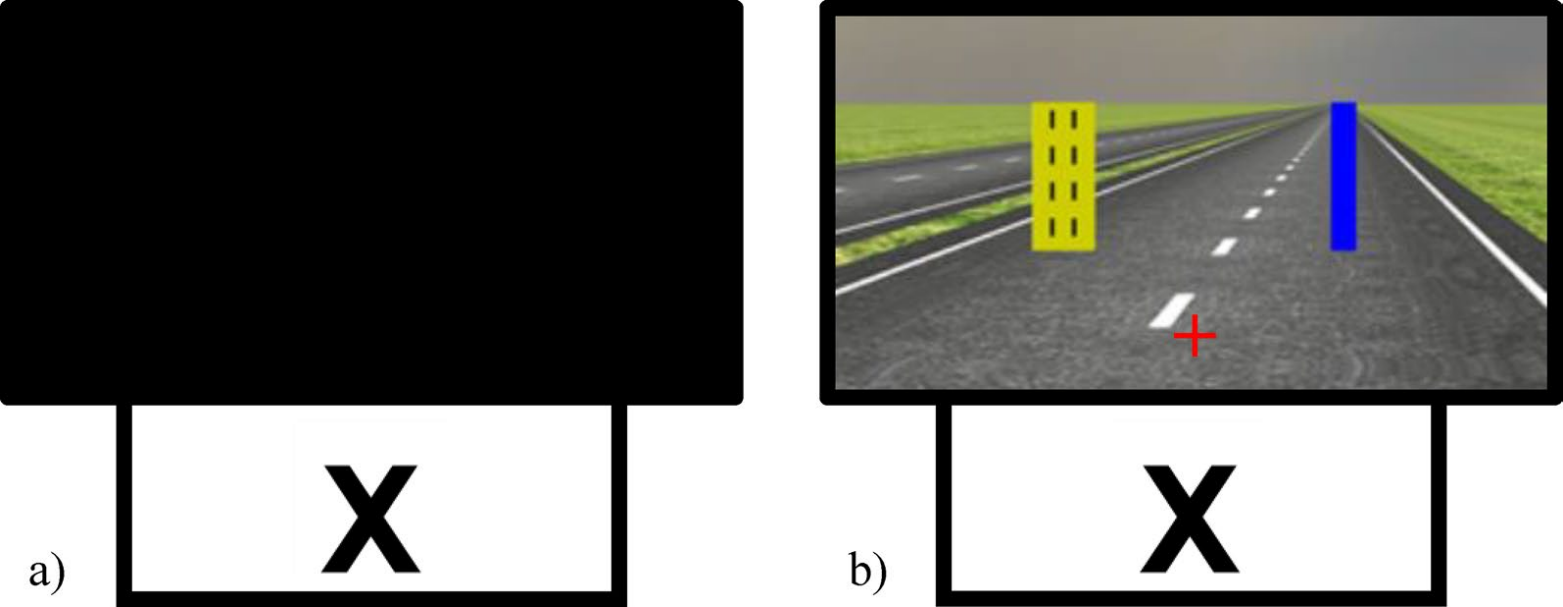
Setup—**a** Session 1, in which the larger top monitor was turned off and the CPT was displayed on the lower monitor; **b** Session 2, in which both monitors displayed their associated tasks. The red cross was not visible to participants during the experiment and is included here as to illustrate participants’ approximate line of sight

The monitors for both tasks were placed approximately two feet directly in front of the participants. The monitor for the CPT task was placed behind and beneath the larger monitor that displayed the driving-based tracking task. For the CPT, the horizontal viewing angle was approximately 31° and the vertical visual angle was approximately 25°; for the tracking task, the horizontal viewing angle was approximately 52° and the vertical visual angle was approximately 40°. During the first experiment session (i.e., Session 1), the larger monitor was turned off so that participants only attended to the CPT displayed on the smaller monitor (Fig. 4a). During the second experiment session (i.e., Session 2), both tasks were simultaneously presented thus requiring participants to split their attention between both monitors as they performed the tasks (Fig. 4b).

The steering wheel used for the tracking task was mounted to the edge of the desk in front of participants. The foot pedal was placed directly in front of participants on the floor at the approximate position their right foot would naturally rest. Participants’ line of sight was located approximately in front of the bottom of the monitor displaying the tracking task, at a point where the CPT and tracking task stimuli were approximately equidistant (red cross, Fig. 4b).

#### Procedure

Before the experiment, participants confirmed valid driver’s licenses and silenced their phones to prevent distractions. After providing informed consent, participants were seated in a testing room and instructed on how to perform the CPT task. They then completed a 120-s practice session to familiarize themselves with the task, including exposure to all three ISI conditions. The first experimental session followed, with the researcher monitoring compliance from outside the room via auditory cues.

Following Session 1, participants received instructions for the dual-task condition and were told to give equal priority to the CPT and tracking tasks. A second 120-s practice session was conducted to acclimate them to the combined task environment. Participants then completed the second experimental session, again with the researcher monitoring task engagement through sound cues from both the pedal and steering wheel.

#### Measures

The independent measures used for analysis included experiment block (1, 2, 3, 4, 5, 6) and ISI condition (1000 ms, 2000 ms, 4000 ms). We collected and/or calculated eight dependent measures in total. From the CPT task we collected five measures: target hit rate (hits), omissions, commissions, correct rejections (CRs), and reaction times (RTs). However, in this CPT variant, omissions and commissions are codependent with hits and CRs, respectively, such that the performance in one measure (e.g., hits) over time is oppositely reflected in the performance of its complementary measure (e.g., omissions). Given this redundancy, we do not analyze omissions here and only utilize commissions for calculating the sensitivity (*d’*) and response bias (*β*) SDT measures. From the driving-based tracking task we collected tracking distance. To relate tracking performance more directly to cognitive demands, we segmented tracking data around critical CPT events in which participants were presented with visual stimuli (targets and non-targets). This allowed us to directly relate fluctuations in tracking performance to concurrent cognitive demands imposed by the CPT.

Table 1 describes these measures as they are used in this experiment.

**Table 1:** Description of measures used in experiment 1

Task	Measure/index	Definition
CPT	Hits (Number of target hits)	Correctly pressing foot pedal when a target is presented
	Omissions (Number of target misses)	Failing to press the foot pedal when a target is presented
	Commissions (Number of false alarms)	Pressing the foot pedal when a non-target is presented
	CRs (Number of Correct Rejections)	Correctly not pressing the foot pedal when a non-target is presented
	RTs (Speed of response to targets)	The time between the onset of a target and the correct pressing of the foot pedal
	*d*’ (Sensitivity)	SDT-based sensitivity (*d*′) for accurately discriminating between targets and non-targets
	*β* (Response bias)	SDT-based response bias (*β*) regarding speed-vs-accuracy tradeoffs
Tracking	Distance (Average distance from target)	Average distance between target and cursor

Note that we do not analyze omissions in this study at all (indicated in gray shading in the table) and only utilize commissions to calculate SDT measures, since both measures are fully codependent with hits and CRs, respectively

#### Data preparation

The CPT data from the practice sessions were discarded, and the CPT data from the first and second experiment sessions were retained for analysis. The data from the driving-based tracking task in the second experiment session, which was stored in a MySQL table, were exported to.csv format for further data processing and analysis in R 4.2.2 (R Core Team, 2012) and R Studio version 1.1.447 (RStudio Team, 2016), which were used for all the analyses reported in this paper.

Prior to statistical analysis, the data files for both the CPT and driving-based tracking tasks were temporally aligned and merged so that tracking performance could be accurately matched with the CPT data. Matching was based on the audio signal (a beep) that OpenDS was programmed to emit to start the CPT task on e-prime through a voice key. Following the alignment, tracking data were filtered to retain only the data from the 500 ms before and after each CPT stimulus presentation. This was done to create matched fixed time windows of tracking data across conditions since the conditions with 4000 ms ISI had twice as much data as the 2000 ms ISI conditions and four times as much data as the 1000 ms ISI conditions (each condition included the same number of CPT targets) (Ballard, 2001). For more fine-grained analysis, the tracking data were further segmented based on whether the corresponding CPT events were targets responded to correctly (hits), non-targets correctly not responded to (CRs), or targets not responded to (commissions), allowing us to assess how tracking performance varied across specific cognitive demands. Because of the low number of omissions, we did not calculate tracking distance averages for trials with these events. Finally, trials with RTs less than 100 ms were removed since they did not likely reflect valid responses (Conners, 2014).

#### Data analysis

We utilized growth curve analyses (GCAs) to analyze the data in our experiments. GCAs are specialized statistical techniques for summarizing longitudinal data with best-fit lines (or smooth curves) that characterize performance trends within observed time windows (Bollen, 2007; Byrne & Crombie, 2003; Kristjansson et al., 2007). This framework is based on building and comparing mixed-effects models that include, in addition to the usual fixed and random effects, fixed terms representing temporal changes of different orders (Baayen et al., 2008; Barr, 2013; Peugh, 2010).

For our GCA, we used the lme4 (Bates et al., 2014) statistical package. The GCAs consisted of terms for linear (i.e., time^1^) and quadratic (i.e., time^2^) time orders, terms for ISI (1000 ms, 2000 ms, 4000 ms), and a random effect of participants on the intercept. All factors included in the models used sum contrast coding (Schad et al., 2020). We attempted to fit more complex random factor terms to the data, but these models did not converge.

For model comparison, we compared four models that increased in complexity:

1. The *Base* model only included baseline time terms and the random factors (with no fixed terms representing ISI or its interaction with any of the time terms) (Model 1, Table A). Note that the Base model included Intercept, Linear, and Quadratic terms/predictors but not any of the fixed factors we manipulated. This means that the Base model could capture the temporal characteristics of the data that were constant across ISI conditions.
2. The *Intercept* model built upon the base model by adding an interaction of ISI with the intercept (Model 2, Table A).
3. The *Linear* model built upon the intercept model by adding the interaction of ISI with the linear (i.e., time^1^) time order (Model 3, Table A).
4. The *Quadratic* model built upon the linear model by adding the interaction of ISI with the quadratic (i.e., time^2^) time order (Model 4, Table A).

We then compared each of these models, using maximum likelihood estimates (Long, 2012) to determine the best time order model to use for the analysis. Following Long’s recommendations, we considered a model to provide a better fit than a simpler model using a *p* <.1 criterion.

In our analysis, we used sum coding, meaning that our factor estimates represent the difference of each level from the grand mean rather than from the third level (Brehm & Alday, 2022). This allows for a more transparent interpretation of model coefficients.

Following Long (2012), we encourage readers to consider the GCA results not only by whether a given measure changes over time, but also by the shape and direction of those changes (e.g., linear vs. curvilinear trends) and how they varied by ISI. We follow these interpretive strategies in the Results and Discussion sections of this and the following experiment, and in the General Discussion, where we synthesize the theoretical implications of the different result patterns across measures, ISIs, and experiments.

### Results

Data from seven participants were removed due to hardware malfunction. Data from the remaining 56 participants (age: *M* = 20.14, *SD* = 1.51) were used for analysis. While this sample size is two participants short of the 58 suggested by our power analysis, this difference is negligible given the overall large number of participants. Of these 56 participants, 39 were female (age: *M* = 20.1, *SD* = 1.33) and 17 were male (age: *M* = 20.2, *SD* = 1.89).

#### Session 1 CPT measures

**Fig. 5:**
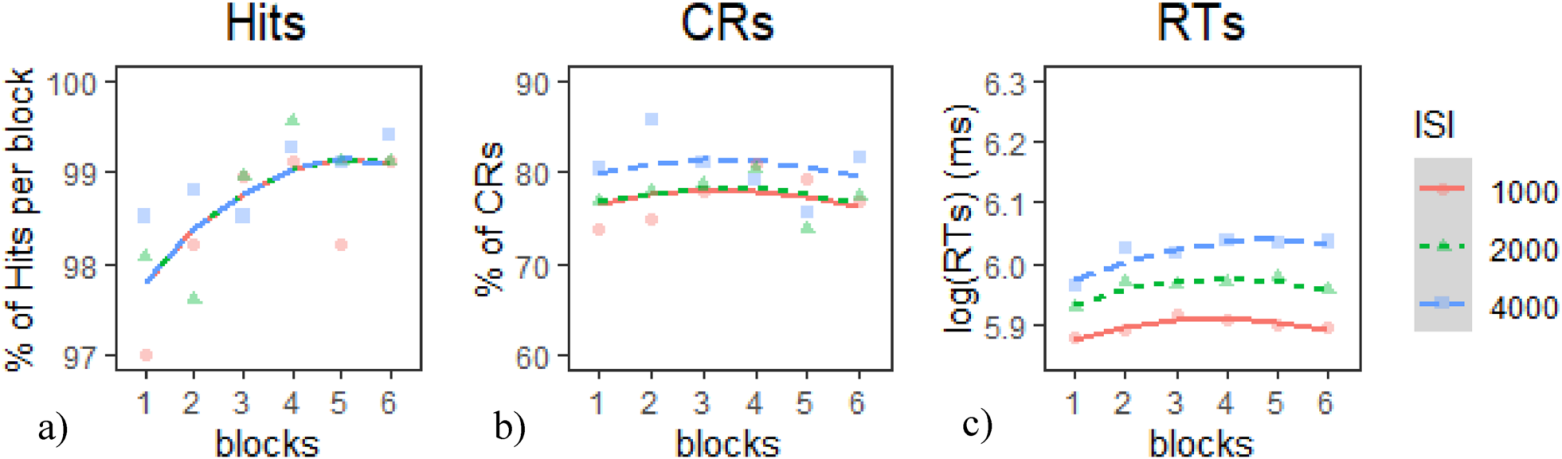
Growth curve analysis graphs for CPT measures in E1 Session 1—**a** hits, **b** CRs, and **c** RTs. Dots represent observed mean performance in each block and ISI combination, and lines represent the chosen model’s predictions

*Target Hits*: None of the models provided a better fit of the data than the Base model according to our criterion. We therefore chose the Base model (Table 2a). Inspection of the model coefficients (Table 3a) and visual inspection of the graph (Fig. 5a) show: 1. a significant positive linear trajectory (i.e., improving performance) across all ISI conditions that tapered off in the last two blocks and 2. no significant differences in performance between ISIs.

**Table 2:** Model comparison tables for CPT measures in E1 session 1—(a) hits, (b) CRs, and (c) RTs

Model	nPar	AIC	BIC	Loglik	Deviance	*Χ*^*2*^	*df*	*p*
*(a) Model comparison* table for hits								
Base	5	6333.4	6358	− 3161.7	6323.4			
Intercept	7	6335.9	6370.3	− 3160.9	6321.9	1.5444	2	0.462
Linear	9	6339.5	6383.8	− 3160.8	6321.5	0.3679	2	0.832
Quadratic	11	6342.6	6396.7	− 3160.3	6320.6	0.9403	2	0.6249
*(b) Model comparison table for CRs*								
Base	5	9151.3	9175.9	− 4570.6	9141.3			
Intercept	7	9150.2	9184.6	− 4568.1	9136.2	5.0606	2	7.96E− 02
Linear	9	9151.2	9195.5	− 4566.6	9133.2	2.9864	2	0.22465
Quadratic	11	9153.7	9207.8	− 4565.9	9131.7	1.499	2	0.4726
*(c) Model comparison table for RTs*								
Base	5	− 1641.6	− 1617	825.8	− 1651.6			
Intercept	7	− 1941.5	− 1907.1	977.8	− 1955.5	303.93	2	< 2e− 16***
Linear	9	− 1943.6	− 1899.4	980.8	− 1961.6	6.1257	2	0.04675*
Quadratic	11	− 1939.8	− 1885.7	980.9	− 1961.8	0.182	2	0.913

* indicates *p* < .05, ** indicates *p* < .01, *** indicates *p* < .001

**Table 3:** Chosen model coefficients tables for CPT measures in E1 session 1—(a) hits, (b) CRs, and (c) RTs

Fixed Effects	Estimate	Std. Error	*df*	*t* value	*p*
(a) Base model coefficients for hits					
(Intercept)	98.7021	0.5492	56	179.725	< 2e−16 ***
time^1^	1.0731	0.402	952	2.67	0.00772 **
time^2^	− 0.4492	0.402	952	− 1.118	0.26404
(b) Intercept model coefficients for CRs					
(Intercept)	78.4392	2.05	56	38.264	< 2e−16***
time^1^	− 0.1423	1.6297	952	− 0.087	0.93
time^2^	− 1.6669	1.6297	952	− 1.023	3.07E−01
ISI1	− 1.1574	0.9409	952	− 1.23	0.219
ISI2	− 0.959	0.9409	952	− 1.019	0.308
(c) Linear model coefficients for RTs					
(Intercept)	5.9600	0.0166	56.0000	358.2630	< 2e−16***
time^1^	0.0271	0.0064	952.0000	4.2540	0.0000***
time^2^	− 0.0270	0.0064	952.0000	− 4.2500	0.0000***
ISI1	− 0.0613	0.0037	952.0000	− 16.6870	< 2e−16***
ISI2	0.0019	0.0037	952.0000	0.5050	0.6136
time^1^:ISI1	− 0.0155	0.0090	952.0000	− 1.7170	0.0863
time^1^:ISI2	− 0.0062	0.0090	952.0000	− 0.6900	0.4906

* indicates *p* < .05, ** indicates *p* < .01, *** indicates *p* < .001

*CRs*: The Intercept model provided the best fit of the data according to our criterion (Table 2b),* χ*^*2*^(2) = 5.06*, p* <.08. Inspection of the model coefficients (Table 3b) and visual inspection of the graph (Fig. 5b) show: 1. no overall temporal trends in the data and 2. no significant differences between ISI conditions.

*RTs*: The linear model provided the best fit of the data according to our criterion (Table 2c),* χ*^*2*^(2) = 6.13*, p* <.05. Inspection of the model coefficients (Table 3c) and visual inspection of the graph (Fig. 5c) show: 1. an overall positive linear trajectory (i.e., degrading performance) during the 4000 ms ISI; 2. an overall negative quadratic trajectory (i.e., declining then tapering off or slightly improving performance) during the 1000 ms and 2000 ms ISIs; and 3. overall fastest (i.e., best) performance during the 1000 ms ISI and slowest (i.e., worst) performance during the 4000 ms ISI.

#### Session 1 SDT indices

**Fig. 6:**
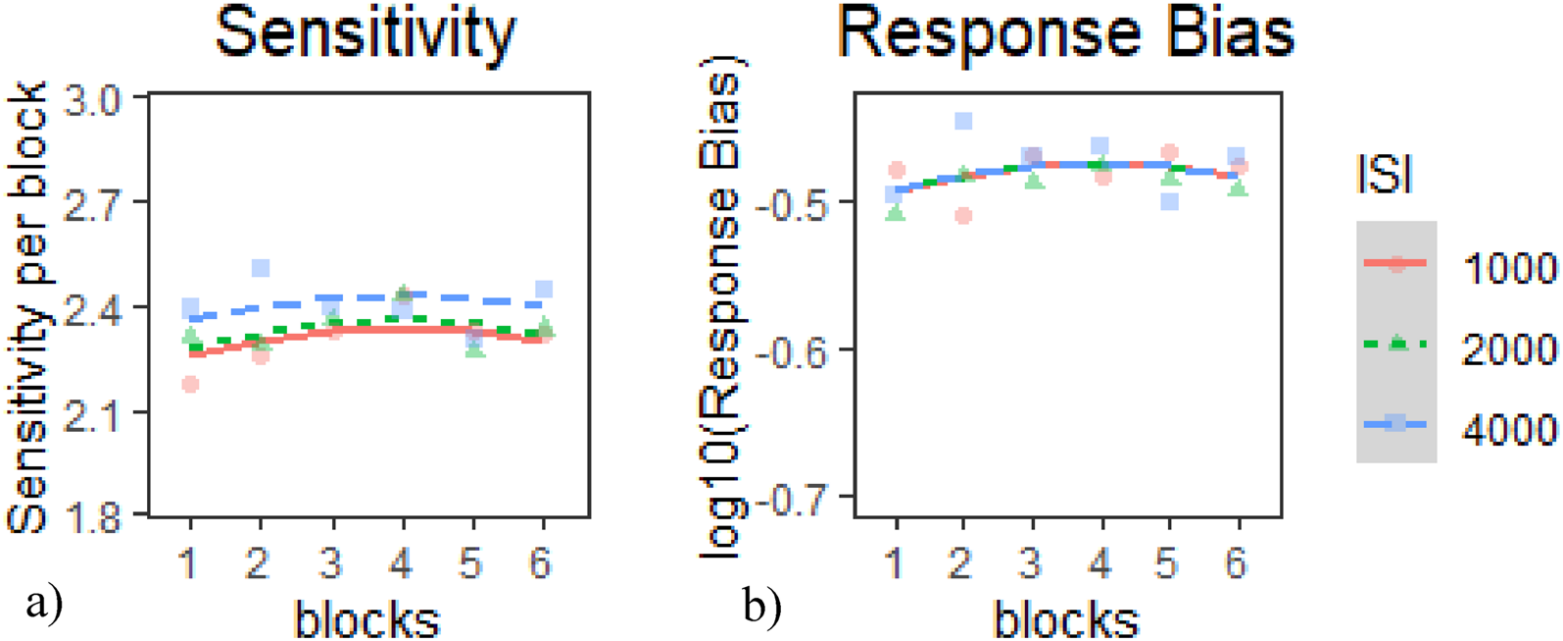
Growth curve analysis graphs for SDT indices in E1 session 1—**a** sensitivity and **b** response bias. Dots represent observed mean performance in each block and ISI combination, and lines represent the chosen model’s predictions

*Sensitivity*: The Intercept model provided the best fit of the data according to our criterion (Table 4a), *χ*^*2*^(2) = 6.03*, p* <.05. Inspection of the model coefficients (Table 5a) and visual inspection of the graph (Fig. 6a) show: 1. no overall temporal trends in the data and 2. marginally worse sensitivity during the 1000 ms and 2000 ms ISI conditions than in the 4000 ms condition.

**Table 4:** Model comparison tables for SDT indices in E1 Session 1—(a) sensitivity and (b) response bias

Model	nPar	AIC	BIC	Loglik	Deviance	*Χ*^*2*^	*df*	*p*
*(a) Model comparison table for sensitivity*								
Base	5	1705.4	1730	− 847.7	1695.4			
Intercept	7	1703.4	1737.8	− 844.69	1689.4	6.0342	2	4.89E−02*
Linear	9	1704.7	1749	− 843.37	1686.7	2.6407	2	0.26705
Quadratic	11	1706.2	1760.3	− 842.11	1684.2	2.5172	2	0.28405
*(b) Model comparison table for response bias*								
Base	5	− 775.57	− 750.99	392.79	− 785.57			
Intercept	7	− 773.05	− 738.64	393.53	− 787.05	1.4819	2	0.4767
Linear	9	− 769.43	− 725.19	393.71	− 787.43	0.375	2	0.829
Quadratic	11	− 766.07	− 712	394.04	− 788.07	0.6456	2	0.7241

* indicates *p* < .05, ** indicates *p* < .01, *** indicates *p* < .001

**Table 5:** Chosen model coefficients tables for SDT indices in E1 session 1—(a) sensitivity and (b) response bias

Fixed effects	Estimate	Std. Error	*df*	t value	*p*
*(a) Intercept model for sensitivity*					
(Intercept)	2.34348	0.05242	56	44.706	< 2e−16***
time^1^	0.03499	0.04047	952	0.864	0.3876
time^2^	− 0.05139	0.04047	952	− 1.27	0.2045
ISI1	− 0.03943	0.02337	952	− 1.687	0.0919
ISI2	− 0.01652	0.02337	952	− 0.707	0.4797
*(b) Base model for response bias*					
(Intercept)	− 0.4814	0.0112	56	− 43.0330	< 2e−16***
Time^1^	0.0075	0.0121	952	0.6210	0.5350
Time^2^	− 0.0119	0.0121	952	− 0.9890	0.3230

* indicates *p* < .05, ** indicates *p* < .01, *** indicates *p* < .001

*Response Bias*: None of the models provided a better fit of the data than the Base model according to our criterion (Table 4b). Inspection of the model coefficients (Table 5b) and visual inspection of the graph (Fig. 6b) show: 1. no overall temporal trends in the data; 2. no significant differences in response bias between ISI conditions; and 3. response bias was indistinguishable in all ISIs and was overall liberal.

#### Session 2 CPT measures

**Fig. 7:**
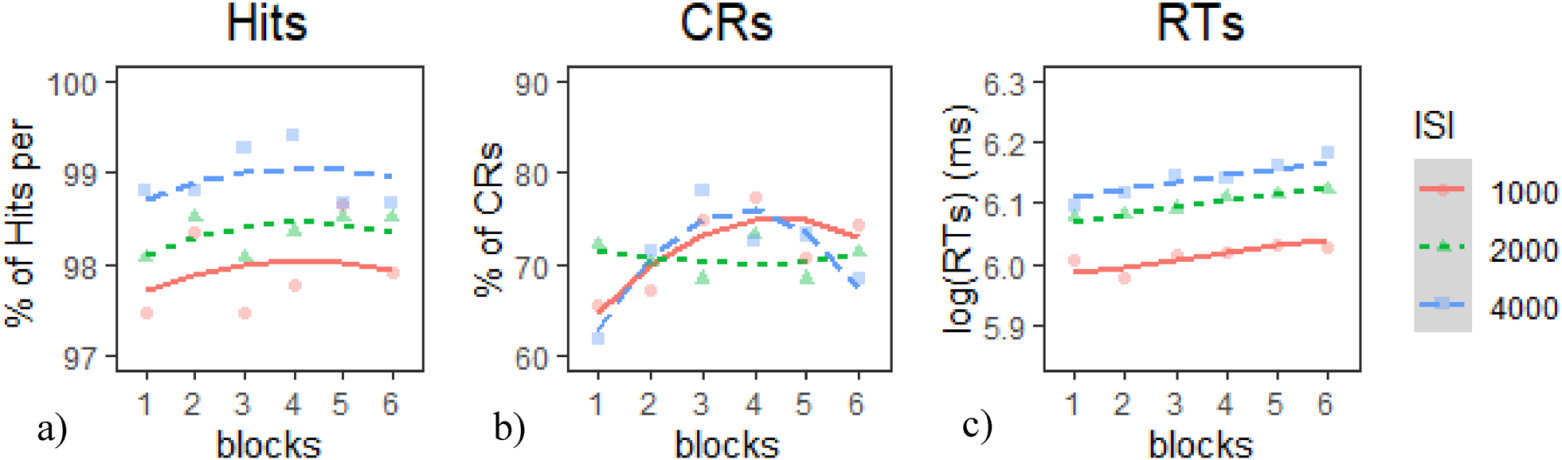
Growth curve analysis graphs for CPT measures in E1 Session 2—**a** hits, **b** CRs, and **c** RTs. Dots represent observed mean performance in each block and ISI combination, and lines represent the chosen model’s predictions

*Target Hits*: The Intercept model provided the best fit of the data according to our criterion (Table 6a), *χ*^*2*^(2) = 9.74*, p* <.01. Inspection of the model coefficients (Table 7a) and visual inspection of the graph (Fig. 7a) show: 1. no overall temporal trends in the data and 2. highest (i.e., best) performance during the 4000 ms ISI condition followed by the 2000 ms ISI condition.

**Table 6:** Model comparison tables for CPT measures in E1 session 2—(a) hits, (b) CRs, and (c) RTs

Model	nPar	AIC	BIC	Loglik	Deviance	*Χ*^*2*^	*df*	*p*
*(a) Model comparison table for hits*								
Base	5	5816.1	5840.7	− 2903.1	5806.1			
Intercept	7	5810.4	5844.8	− 2898.2	5796.4	9.7378	2	0.007682**
Linear	9	5813.9	5858.1	− 2897.9	5795.9	0.5217	2	0.770386
Quadratic	11	5817.3	5871.4	− 2897.7	5795.3	0.5115	2	0.774321
*(b) Model comparison table for CRs*								
Base	5	9324.6	9349.2	− 4657.3	9314.6			
Intercept	7	9328.2	9362.6	− 4657.1	9314.2	0.4054	2	8.17E−01
Linear	9	9329.3	9373.5	− 4655.6	9311.3	2.8876	2	0.23603
Quadratic	11	9325.9	9380	− 4652	9303.9	7.3421	2	0.02545*
*(c) Model comparison table for RTs*								
Base	5	− 1422.6	− 1398	716.29	− 1432.6			
Intercept	7	− 1692	− 1657.5	852.97	− 1706	273.36	2	
Linear	9	− 1692.3	− 1648	855.15	− 1710.3	4.3476	2	
Quadratic	11	− 1688.5	− 1634.4	855.25	− 1710.5	0.2049	2	

* indicates *p* < .05, ** indicates *p* < .01, *** indicates *p* < .001

**Table 7:** Chosen model coefficients tables for CPT measures in E1 session 2—(a) hits, (b) CRs, and (c) RTs

Fixed effects	Estimate	Std. error	*df*	*t* value	*p*
*(a) Intercept model coefficients for hits*					
(Intercept)	98.40443	0.24992	56	393.751	< 2e−16***
time^1^	0.19565	0.31921	952	0.613	0.5401
time^2^	− 0.20566	0.31921	952	− 0.644	0.5195
ISI1	− 0.46296	0.1843	952	− 2.512	0.0122*
ISI2	− 0.06614	0.1843	952	− 0.359	0.7198
*(b) Quadratic model coefficients for CRs*					
(Intercept)	71.0979	2.4254	56	29.314	< 2e−16***
time^1^	3.4624	1.7623	952	1.965	0.04974*
time^2^	− 4.7843	1.7623	952	− 2.715	0.00675**
ISI1	0.6283	1.0175	952	0.618	5.37E−01
ISI2	− 0.463	1.0175	952	− 0.455	0.6492
time^1^:ISI1	3.4387	2.4923	952	1.38	0.16799
time^1^:ISI2	− 3.8892	2.4923	952	− 1.561	0.11897
time^2^:ISI1	− 0.4763	2.4923	952	− 0.191	0.84849
time^2^:ISI2	6.0833	2.4923	952	2.441	0.01483*
*(c) Intercept model coefficients for RTs*					
(Intercept)	6.0839	0.0189	60	322.224	< 2e−16***
time^1^	0.0468	0.0072	952	6.4820	0.0000***
time^2^	− 0.0001	0.0072	952	− 0.0140	0.9885
ISI1	− 0.0704	0.0042	952	− 16.8770	< 2e−16***
ISI2	0.0148	0.0042	952	3.5550	0.0004***

* indicates *p* < .05, ** indicates *p* < .01, *** indicates *p* < .001

*CRs*: The quadratic model provided the best fit of the data according to our criterion (Table 6b),* χ*^*2*^(2) = 7.34*, p* <.03. Inspection of the model coefficients (Table 7b) and visual inspection of the graph (Fig. 7b) show: 1. an overall positive linear trajectory (i.e., improving performance); 2. a negative quadratic trajectory (i.e., improving then degrading performance) in the 1000 ms and 4000 ms IS conditions; and 3. a flat trajectory (i.e., constant performance) in the 2000 ms ISI.

*RTs*: The intercept model provided the best fit of the data according to our criterion (Table 6c),* χ*^*2*^(2) = 273.36*, p* <.001. Inspection of the model coefficients (Table 7c) and visual inspection of the graph (Fig. 7c) show: 1. positive linear trajectories (i.e., worsening performance) across all ISI conditions and 2. significantly faster (i.e., better) performance in the 1000 ms ISI condition than in the 2000 and 4000 ms ISI conditions.

#### Session 2 SDT indices

**Fig. 8:**
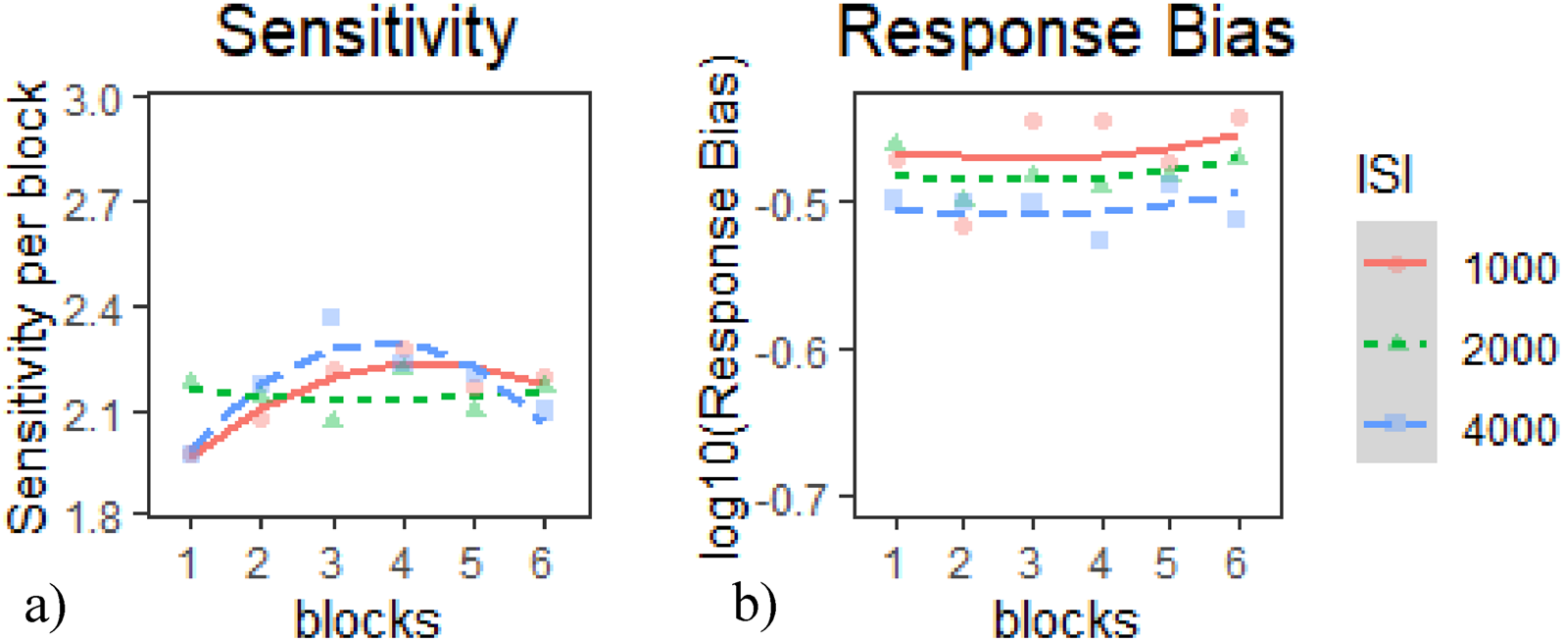
Growth curve analysis graphs for SDT indices in E1 Session 2—**a** sensitivity and **b** response bias. Dots represent observed mean performance in each block and ISI combination, and lines represent the chosen model’s predictions

*Sensitivity*: The quadratic model provided the best fit of the data according to our criterion (Table 8a), *χ*^*2*^(2) = 7.89*, p* <.02. Inspection of the model coefficients (Table 9a) and visual inspection of the graph (Fig. 8a) show: 1. a negative quadratic trajectory (i.e., increasing then declining sensitivity) in the 1000 ms and the 4000 ms ISI conditions; and 2. a flat trajectory (constant sensitivity) in the 2000 ms ISI.

**Table 8:** Model comparison tables for SDT indices in E1 session 2—(a) sensitivity and (b) response bias

Model	nPar	AIC	BIC	Loglik	Deviance	*Χ*^*2*^	*df*	*p*
*(a) Model comparison table for sensitivity*								
Base	5	1877	1901.6	− 933.5	1867			
Intercept	7	1880.5	1914.9	− 933.26	1866.5	0.4783	2	0.7873
Linear	9	1882	1926.2	− 931.98	1864	2.5564	2	0.27854
Quadratic	11	1878.1	1932.2	− 928.04	1856.1	7.8915	2	0.01934*
*(b) Model comparison table for response bias*								
Base	5	− 632.1	− 607.52	321.05	− 642.1			
Intercept	7	− 637.11	− 602.7	325.55	− 651.11	9.0079	2	0.01107*
Linear	9	− 634.82	− 590.58	326.41	− 652.82	1.7127	2	0.4247
Quadratic	11	− 631.57	− 577.5	326.79	− 653.57	0.7521	2	0.68658

* indicates *p* < .05, ** indicates *p* < .01, *** indicates *p* < .001

**Table 9:** Chosen model coefficients tables for SDT indices in E1 session 1—(a) sensitivity and (b) response bias

Fixed effects	Estimate	Std. error	*df*	t value	*p*
*(a) Quadratic model coefficients for sensitivity*					
(Intercept)	2.153712	0.060536	55.999996	35.577	< 2e−16***
time^1^	0.07745	0.043806	952.000001	1.768	0.07738
time^2^	− 0.122764	0.043806	952.000001	− 2.802	0.00517**
ISI1	− 0.004099	0.025291	952.000001	− 0.162	0.87127
ISI2	− 0.012764	0.025291	952.000001	− 0.505	0.6139
time^1^:ISI1	0.090254	0.061951	952.000001	1.457	0.14548
time^1^:ISI2	− 0.081461	0.061951	952.000001	− 1.315	0.18885
time^2^:ISI1	− 0.023175	0.061951	952.000001	− 0.374	0.70842
time^2^:ISI2	0.161276	0.061951	952.000001	2.603	0.00938**
*(b) Intercept model coefficients for response bias*					
(Intercept)	− 0.484496	0.008131	56	− 59.587	< 2e−16***
time^1^	0.0088	0.0132	952.000001	0.6670	0.5050
time^2^	0.0085	0.0132	952.000001	0.6420	0.5210
ISI1	0.0186	0.0076	952.000001	2.4360	0.0150
ISI2	0.0024	0.0076	952.000001	0.3110	0.7560

* indicates *p* < .05, ** indicates *p* < .01, *** indicates *p* < .001

*Response Bias*: The intercept model provided the best fit of the data according to our criterion (Table 8b), *χ*^*2*^(2) = 9.01*, p* <.02. Inspection of the model coefficients (Table 9b) and visual inspection of the graph (Fig. 8b) show: 1. a flat trajectory (i.e., constant performance) during all ISI conditions and 2. response bias across all ISI conditions was overall liberal, and most liberal during the 4000 ms ISI condition.

#### Session 2 tracking measure

**Fig. 9:**
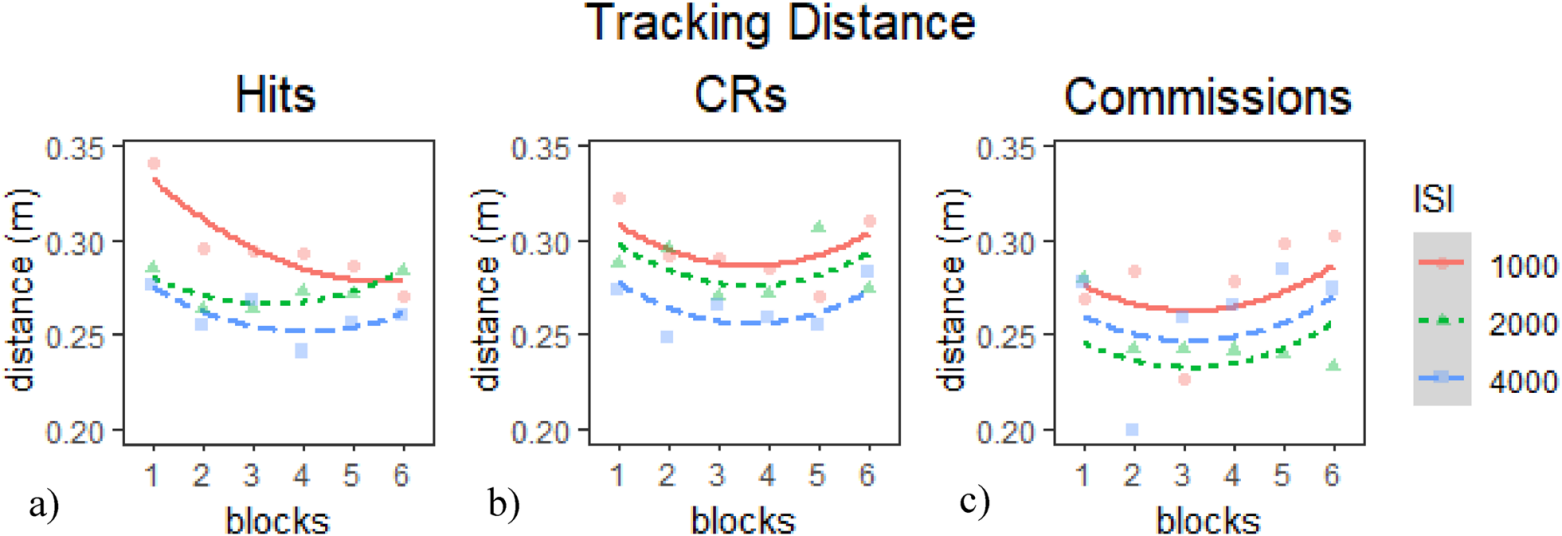
Growth curve analysis graphs for tracking distance measures in E1 session 2—**a** hits, **b** CRs, and **c** commissions. Dots represent observed mean performance in each block and ISI combination, and lines represent the chosen model’s predictions

*Average Tracking Distance during Hits*: The linear model provided the best fit of the data according to our criterion (Table 10a), *χ*^*2*^(2) = 10.42*, p* <.01. Inspection of the model coefficients (Table 11a) and visual inspection of the graph (Fig. 9a) show: 1. a positive quadratic trajectory (i.e., rapid improvement in earlier block and slower improvement or deterioration in later blocks) in all ISI conditions; 2) a negative linear trajectory (i.e., improving performance) throughout the session in the 1000 ms ISI condition, but in the 2000 ms and 4000 ms ISIs this trajectory was qualified by worsening performance in later blocks; and 3. overall worse performance during the 1000 ms ISI than during the 2000 ms and 4000 ms ISIs.

**Table 10:** Model comparison tables for tracking distance measures in E1 session 2—(a) hits, (b) CRs, and (c) commissions

Model	nPar	AIC	BIC	Loglik	Deviance	*Χ*^*2*^	*df*	*p*
*(a) Model comparison table for tracking distance during hits*								
Base	5	− 2002.5	− 1978	1006.3	− 2012.5			
Intercept	7	− 2033.8	− 1999.4	1023.9	− 2047.8	35.2418	2	2.23E−08***
Linear	9	− 2040.2	− 1996	1029.1	− 2058.2	10.4216	2	0.005457**
Quadratic	11	− 2036.3	− 1982.2	1029.1	− 2058.3	0.0701	2	0.965542
*(b) Model comparison table for tracking distance during CRs*								
Base	5	− 1226.9	− 1202.5	618.47	− 1236.9			
Intercept	7	− 1233.6	− 1199.3	623.78	− 1247.6	10.6232	2	4.93E−03**
Linear	9	− 1230.5	− 1186.4	624.25	− 1248.5	0.9497	2	0.621988
Quadratic	11	− 1228	− 1174.1	624.97	− 1250	1.4404	2	0.486663
*(c) Model comparison table for tracking distance during commissions*								
Base	5	− 677.42	− 654.24	343.71	− 687.42			
Intercept	7	− 678.63	− 646.18	346.31	− 692.63	5.21	2	7.39E−02*
Linear	9	− 678.53	− 636.82	348.26	− 696.53	3.9019	2	0.1421
Quadratic	11	− 674.71	− 623.73	348.36	− 696.71	0.1858	2	0.9113

* indicates *p* < .05, ** indicates *p* < .01, *** indicates *p* < .001

**Table 11:** Chosen model coefficients tables for tracking distance measures in E1 session 2—(a) hits, (b) CRs, and (c) commissions

Fixed effects	Estimate	Std. error	*df*	*t* value	*P*
*(a) Linear model coefficients for tracking distance during hits*					
(Intercept)	2.77E− 01	1.05E− 02	5.60E + 01	26.358	< 2e− 16***
time^1^	− 1.81E− 02	6.22E− 03	9.52E + 02	− 2.909	0.00371**
time^2^	1.56E− 02	6.22E− 03	9.52E + 02	2.509	1.23E− 02*
ISI1	2.01E− 02	3.59E− 03	9.52E + 02	5.603	2.75E− 08***
ISI2	− 3.18E− 03	3.59E− 03	9.52E + 02	− 0.885	3.76E− 01
time^1^:ISI1	− 2.72E− 02	8.79E− 03	9.52E + 02	− 3.09	2.06E− 03**
time^1^:ISI2	0.020918	0.00879	952.000001	2.38	0.01752*
*(b) Intercept model coefficients for tracking distance during CRs*					
(Intercept)	2.82E− 01	1.05E− 02	5.60E + 01	26.936	< 2e− 16***
time^1^	− 3.87E− 03	9.49E− 03	9.33E + 02	− 0.408	0.6835
time^2^	1.93E− 02	9.48E− 03	9.33E + 02	2.037	0.042*
ISI1	1.37E− 02	5.50E− 03	9.34E + 02	2.483	1.32E−02*
ISI2	3.20E− 03	5.47E− 03	9.33E + 02	0.584	5.59E−01
*(c) Intercept model coefficients for tracking distance during commissions*					
(Intercept)	2.56E− 01	1.04E− 02	5.38E + 01	24.526	< 2e− 16***
time^1^	8.74E− 03	1.29E− 02	7.11E + 02	0.675	0.4997
time^2^	1.80E− 02	1.31E− 02	7.13E + 02	1.376	0.1692
ISI1	1.53E− 02	7.56E− 03	7.13E + 02	2.024	4.33E− 02*
ISI2	− 1.46E− 02	7.54E− 03	7.15E + 02	− 1.932	5.38E− 02

* indicates *p* < .05, ** indicates *p* < .01, *** indicates *p* < .001

*Average Tracking Distance during CRs*: The intercept model provided the best fit of the data according to our criterion (Table 10b), *χ*^*2*^(2) = 10.62*, p* <.01. Inspection of the model coefficients (Table 11b) and visual inspection of the graph (Fig. 9b) show: 1. positive quadratic trajectories (i.e., improving then declining performance) during all ISI conditions; and 2. overall worse performance during the 1000 ms ISI than during the 2000 ms and 4000 ms ISIs.

*Average Tracking Distance during Commissions*: The intercept model provided the best fit of the data according to our criterion (Table 10c), *χ*^*2*^(2) = 5.21*, p* <.08. Inspection of the model coefficients (Table 11c) and visual inspection of the graph (Fig. 9c) show: 1. no overall significant temporal trends in the data and 2. overall worse performance during the 1000 ms ISI than during the 2000 ms and 4000 ms ISIs.

### Discussion

With respect to the first part of our first question for this experiment, which was whether performance would change over time for each of the measures and indices associated with the CPT and tracking tasks, we note that some, but not all measures showed different performance trajectories across the session time-blocks. With respect to our second question, which was whether the temporal trajectories of any of our measures or indices would differ when target presentation rates are faster compared to when they are slower, we note that again, some, but not all measures showed different performance trajectories in the different ISI conditions.

Although our primary interest lies in the dual-task Session 2, results from the single-task Session 1 help validate our CPT paradigm. In Session 1, hits showed a consistent practice effects in all ISIs (Fig. 1c—dashed line). CRs were generally worse in the 1000 ms and 2000 ms ISIs than in the 4000 ms ISI but remained stable over time (Fig. 1a). RTs showed both linear and curvilinear performance across ISIs. Specifically, in the 1000 ms and 2000 ms RTs became slightly slower over the next few blocks and then slightly improved in the later blocks (Fig. 1d—solid line). In the 4000 ms ISI, performance became slower across the entire session (Fig. 1b—solid line). As for the SDT indices, sensitivity was worse in the 1000 ms and 2000 ms ISIs than in the 4000 ms ISI but remained stable over time, while response bias remained consistently liberal. Overall, these results suggest that the CPT in Session 1 was sensitive enough to detect some temporal performance changes (e.g., practice effects in hits) and vigilance decrements (progressively slowing RTs in the 4000 ms ISI). This was especially evident in how the ISI manipulation modulated RTs and revealed patterns aligned with underload-related decline. However, the task in Session 1 may not have been sufficiently demanding to elicit effects across all measures, as performance was dominated by extended practice effects. This limitation motivated the design of Session 2, where prior CPT experience and the added cognitive demands of multitasking were expected to produce clearer and more widespread patterns of performance changes.

In Session 2, performance on both CPT and tracking tasks also showed a mix of practice effects and vigilance decrements. Hits were relatively flat across ISIs, with overall worse performance in the 1000 ms ISI, suggesting that this high-demand condition taxed participants throughout. CRs followed curvilinear trajectories in the 1000 ms and 400 ms ISIs, indicating initial practice gains followed by vigilance decrements, while remaining stable in the 2000 ms ISI. RTs were the only CPT measure to show consistent vigilance decrements across all ISIs, with a steady decline in speed from the earliest blocks onward (Fig. 1b—solid line). Regarding the SDT indices, sensitivity mirrored performance in the CRs measure, with curvilinear trajectories in the 1000 ms and 4000 ms ISI, and flat performance in the 2000 ms ISI; and response bias remained constantly liberal during the session.

Tracking distance showed more nuanced patterns. During hits, tracking in the 1000 ms ISI condition progressively improved across the session, though gains slowed in later blocks. In the 2000 ms and 4000 ms ISIs, tracking showed mixed practice and vigilance decrement effects. In contrast, tracking during CRs followed a curvilinear trajectory across all ISIs consistent with early practice effects followed by later vigilance decrements, while tracking during Commissions remained relatively flat, indicating performance stability not strongly affected by either learning or fatigue. Notably, differences in tracking performance across ISIs during hits were not mirrored by corresponding differences in CPT Hit rates.

Overall, vigilance decrements in the CPT were most evident in declining CRs and sensitivity (in the 1000 ms and 4000 ms ISIs), and in RTs (across all ISIs). In the tracking task, vigilance decrements emerged primarily in the 2000 ms and 4000 ms ISIs during both hits and CR trials, though not during Commissions. These patterns suggest that under high cognitive demand, participants prioritized maintaining accurate target detection in the CPT task, particularly in the more time-pressured ISI conditions, even as performance on the concurrent tracking task declined.

Furthermore, the vigilance decrements observed in the 1000 ms and 4000 ms ISIs for CRs and sensitivity suggest that, in these two ISI conditions, despite their general preference for speed over accuracy (as indicated by response bias), participants prioritized responding accurately to true targets at the expense of inhibiting responses to non-targets. This may initially seem to suggest that maintaining a stable rate of target hits in these two ISI conditions similarly taxed executive resources. However, differences in the other measures between these two ISI conditions suggest that the similar temporal patterns in CRs and sensitivity are driven by different underlying mechanisms. In the 1000 ms ISI, as cognitive load increased, participants likely preserved target hit rates at the expense of CRs, leading to reduced sensitivity. This was also reflected in the longer practice effect in tracking distance during hits and in overall faster and less accurate responses in the 1000 ms ISI than in the other ISI conditions. In contrast, in the 4000 ms ISI condition, it is more likely that decreasing arousal may have caused greater difficulty in CRs, which is consistent with the overall higher hit accuracy and better tracking performance in this ISI condition, and with the linear decrease in RTs across blocks in all ISIs. Interestingly, performance appeared most stable in the 2000 ms ISI, suggesting an optimal balance of arousal and task demand avoiding both overload and under engagement (Wiener et al., 1984). To help the reader understand the overall pattern of results, Appendix B includes a summary table of the findings and their theoretical implications.

These findings highlight that vigilance decrements in E1 did not stem from a single source, but rather reflected multiple contributing mechanisms that varied across measures and ISI conditions. Building on this interpretation, Experiment 2 was designed to address two limitations. First, practice effects may have masked vigilance decrements in Session 2, as participants did not complete the tracking task in isolation (i.e., as the single task) beforehand. Second, the inclusion of three ISIs may have reduced statistical power to detect differences between the shortest and longest ISI conditions.

## Experiment 2

The purpose of Experiment 2 (E2) was to test whether the results of E1 can be replicated and extended after addressing the two concerns about E1. Specifically, we made two changes to the paradigm in E2. First, we included an additional practice session to better acclimate participants to the tracking task and therefore increase the likelihood of observing vigilance decrements. Second, we removed the 2000 ms ISI condition to increase power. This experiment therefore addressed the same questions: whether and how performance would change across the six blocks for each of the specific measures associated with the CPT and tracking tasks, and how would the temporal trajectories of any of our measures differ when target presentation rates are faster compared to when they are slower? In particular, we asked whether the different patterns of vigilance effects in the 1000 ms and 4000 ms conditions will hold in this more powerful design.

### Methods

We made two key changes to the E1 paradigm. First, we added a practice session in which participants performed the driving-based tracking task in the absence of the CPT for approximately 30 s. This new practice session occurred after participants completed the first experiment session, and before they began the CPT practice session for the second experiment session. Second, we removed the 2000 ms ISI condition. As a result, there were more trials (20 instead of 15) in each of the two remaining ISIs: 1000 ms and 4000 ms. This resulted in increased power as well as fewer trials per block (40 instead of 45) and per experiment session (240 instead 270) in comparison with E1. Although this also reduced the overall time of each experiment session (from approximately 12 to approximately 11 min), this overall reduction in overall duration is not likely to affect the observation of vigilance decrements, given that these effects were observed much earlier in E1. All other methods in E2 (i.e., participant selection criteria, hardware and software, tasks, setup, data preparation, and data analysis) were the same as in E1.

### Results

#### Session 1 CPT measures

**Fig. 10:**
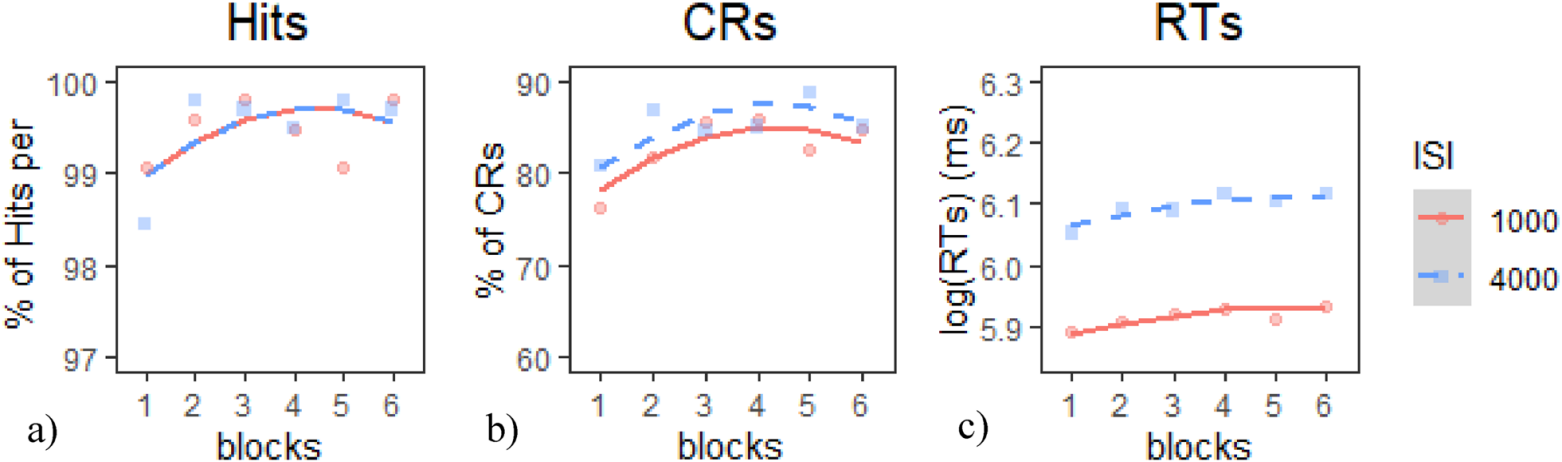
Growth curve analysis graphs for CPT measures in E2 Session 1—**a** hits, **b** CRs, and **c** RTs. Dots represent observed mean performance in each block and ISI combination, and lines represent the chosen model’s predictions

*Target Hits*: None of the models provided a better fit of the data than the base model according to our criterion; therefore, we chose to use the Base model (Table 12a). Inspection of the model coefficients (Table 13a) and visual inspection of the graph (Fig. 10a) show: 1. a positive linear trajectory (i.e., improving performance) during both ISI conditions that tapered off in the last two blocks and 2. no significant differences in performance between ISIs.

**Table 12:** Model comparison tables for CPT measures in E2 session 1—(a) hits, (b) CRs, and (c) RTs

Model	nPar	AIC	BIC	Loglik	Deviance	*Χ*^*2*^	*df*	*p*
*(a) Model comparison table for hits*								
Base	5	3392.4	3415.3	− 1691.2	3382.4			
Intercept	6	3394.4	3421.9	− 1691.2	3382.4	0.0086	1	9.26E−01
Linear	7	3395.2	3427.3	− 1690.6	3381.2	1.2438	1	0.2647
Quadratic	8	3396.2	3432.8	− 1690.1	3380.2	0.9883	1	0.3202
*(b) Model comparison table for CRs*								
Base	5	6287.2	6310.1	− 3138.6	6277.2			
Intercept	6	6285.8	6313.2	− 3136.9	6273.8	3.4615	1	0.06281
Linear	7	6287.4	6319.4	− 3136.7	6273.4	0.4083	1	0.52281
Quadratic	8	6288.9	6325.5	− 3136.4	6272.9	0.4864	1	0.48555
*(c) Model comparison table for RTs*								
Base	5	− 803.48	− 780.58	406.74	− 813.48			
Intercept	6	− 1336.42	− 1308.95	674.21	− 1348.42	534.95	1	< 2e−16***
Linear	7	− 1335.72	− 1303.66	674.86	− 1349.72	1.2935	1	0.2554
Quadratic	8	− 1333.98	− 1297.34	674.99	− 1349.98	0.2602	1	0.61

* indicates *p* < .05, ** indicates *p* < .01, *** indicates *p* < .001

**Table 13:** Chosen model coefficients tables for CPT measures in E2 session 1—(a) hits, (b) CRs, and (c) RTs

Fixed effects	Estimate	Std. error	*df*	*t* value	*p*
*(a) Base model coefficients for hits*					
(Intercept)	99.4705	0.106	60	938.81	< 2e−16***
time^1^	0.4669	0.229	660	2.039	0.0418*
time^2^	− 0.3864	0.229	660	− 1.688	0.0919
*(b) Intercept model coefficients for CRs*					
(Intercept)	83.9236	1.6123	60	52.052	< 2e−16***
time^1^	4.3078	1.5979	660	2.696	7.20E−03**
time^2^	− 3.7961	1.5979	660	− 2.376	0.0178*
ISI1	− 1.2153	0.6523	660	− 1.863	0.0629
*(c) Intercept model coefficients for RTs*					
(Intercept)	6.0059	0.0156	60	386.15	< 2e−16***
time^1^	0.0365	0.0076	660	4.827	1.73E−06***
time^2^	− 0.0145	0.0076	660	− 1.918	0.0555
ISI1	− 0.0887	0.0031	660	− 28.712	< 2e−16***

* indicates *p* < .05, ** indicates *p* < .01, *** indicates *p* < .001

*CRs*: The Intercept model provided the best fit of the data according to our criterion (Table 12b), *χ*^*2*^(2) = 3.46*, p* <.07. Inspection of the model coefficients (Table 13b) and visual inspection of the graph (Fig. 10b) show: 1. a positive linear trajectory (i.e., improving performance) during both ISI conditions that tapered off in the last two blocks (which was supported by both significant linear and quadratic model terms) and 2. lower (i.e., worse) performance during the 1000 ms ISI.

*RTs*: The Intercept model provided the best fit of the data according to our criterion (Table 12c),* χ*^*2*^(1) = 534.95*, p* <.001. Inspection of the model coefficients (Table 13c) and visual inspection of the graph (Fig. 10c) show: 1. positive linear trajectories (i.e., worsening performance) across both ISI conditions and 2. faster (i.e., better) performance during the 1000 ms ISI.

#### Session 1 SDT indices

**Fig. 11:**
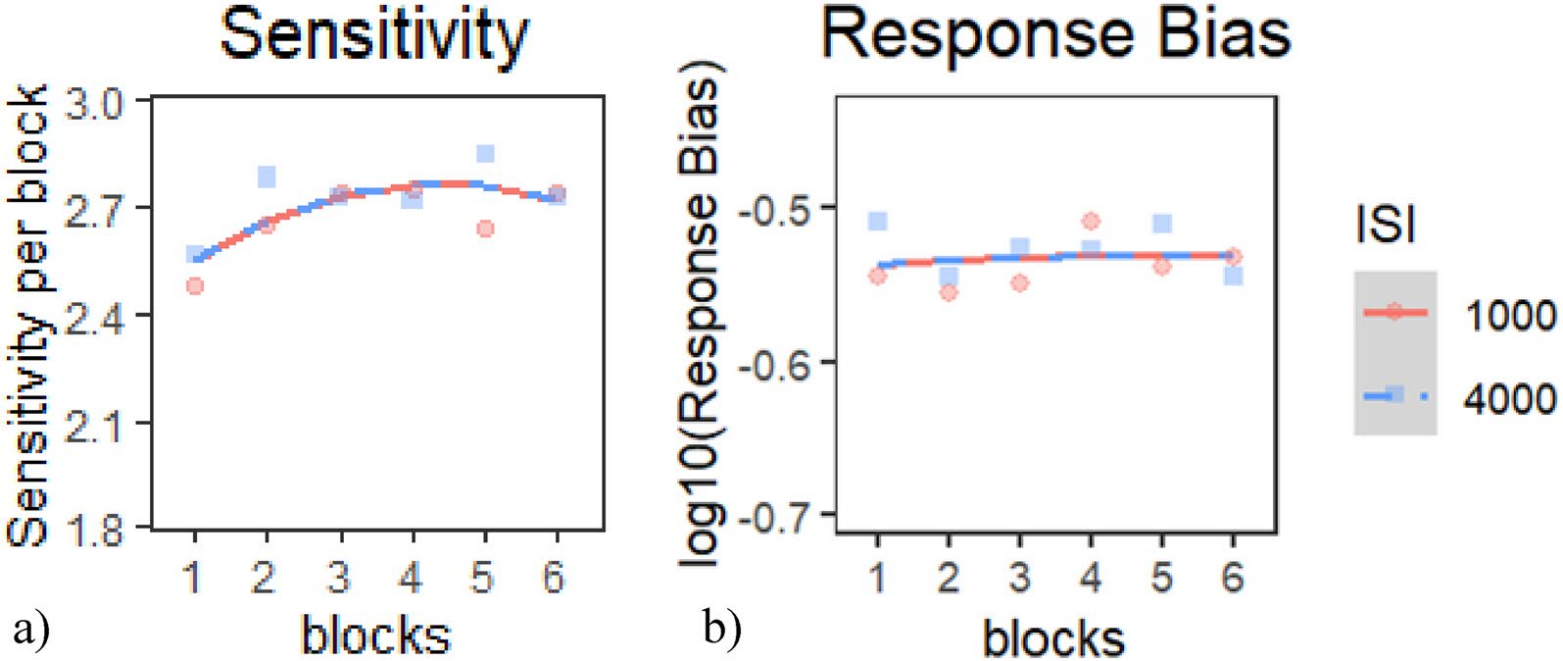
Growth curve analysis graphs for SDT indices in E2 Session 1—**a** sensitivity and **b** response bias. Dots represent observed mean performance in each block and ISI combination, and lines represent the chosen model’s predictions

*Sensitivity*: None of the models provided a better fit of the data than the base model according to our criterion; therefore, we chose to use the Base model (Table 14a). Inspection of the model coefficients (Table 15a) and visual inspection of the graph (Fig. 11a) show: 1. positive linear trajectories combined with negative quadratic trends (i.e., improving sensitivity at a faster rate in earlier blocks than in later one) during both ISI conditions and 2. no differences in performance between ISIs.

**Table 14:** Model comparison tables for SDT indices in E2 session 1—(a) sensitivity and (b) response bias

Model	nPar	AIC	BIC	Loglik	Deviance	*Χ*^*2*^	*df*	*p*
*(a) Model comparison table for sensitivity*								
Base	5	1125.9	1148.8	− 557.96	1115.9			
Intercept	6	1125.2	1152.7	− 556.62	1113.2	2.6892	1	0.101
Linear	7	1127.1	1159.1	− 556.53	1113.1	0.1806	1	0.6708
Quadratic	8	1129	1165.6	− 556.47	1113	0.1067	1	0.7439
*(b) Model comparison table for response bias*								
Base	5	− 492.68	− 469.78	251.34	− 502.68			
Intercept	6	− 491.58	− 464.11	251.79	− 503.58	0.9058	1	0.3412
Linear	7	− 490.54	− 458.49	252.27	− 504.54	0.9618	1	0.3267
Quadratic	8	− 488.59	− 451.95	252.29	− 504.59	0.0422	1	0.8373

**Table 15:** Chosen model coefficients tables for SDT indices in E2 session 1—(a) sensitivity and (b) response bias

Fixed effects	Estimate	Std. error	d*f*	*t* value	*p*
*(a) Base model coefficients for sensitivity*					
(Intercept)	2.69149	0.04251	60	63.307	< 2e−16***
time^1^	0.1376	0.0447	660.0000	3.0800	0.0022**
time^2^	− 0.1124	0.0447	660.0000	− 2.5160	0.0121*
*(b) Base model coefficients for response bias*					
(Intercept)	− 0.5325	0.0116	60.0000	− 46.0670	< 2e−16***
time^1^	0.0047	0.0148	660.0000	0.3160	0.7520
time^2^	− 0.0029	0.0148	660.0000	− 0.1980	0.8430

* indicates *p* < .05, ** indicates *p* < .01, *** indicates *p* < .001

*Response Bias*: None of the models provided a better fit of the data than the base model according to our criterion, and therefore, we chose to use the Base model (Table 14b). Inspection of the model coefficients (Table 15b) and visual inspection of the graph (Fig. 11b) show: 1. a flat trajectory (i.e., constant performance) during all ISI conditions; 2. response bias across all ISIs was overall liberal; and 3. there were no significant differences in response bias between ISIs.

#### Session 2 CPT measures

**Fig. 12:**
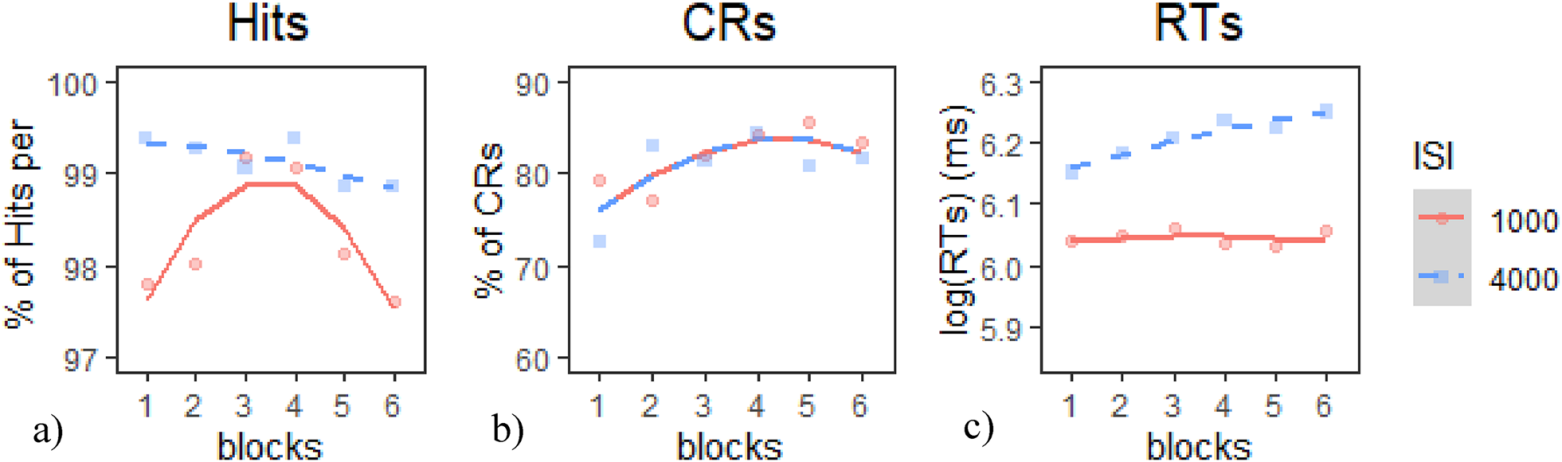
Growth curve analysis graphs for CPT measures in E2 Session 2—**a** hits, **b** CRs, and **c** RTs. Dots represent observed mean performance in each block and ISI combination, and lines represent the chosen model’s predictions

*Target Hits*: The Quadratic model provided the best fit of the data according to our criterion (Table 16a), *χ*^*2*^(1) = 3.75*, p* <.06. Inspection of the model coefficients (Table 17a) and visual inspection of the graph (Fig. 12a) show: 1. a flat trajectory (i.e., constant performance) during the 4000 ms ISI condition; 2. a negative quadratic trajectory (i.e., improving then declining performance) during the 1000 ms ISI; and 3. highest (i.e., better) performance during the 4000 ms ISI.

**Table 16:** Model comparison tables for CPT measures in E2 Session 2—(a) hits, (b) CRs, and (c) RTs

Model	nPar	AIC	BIC	Loglik	Deviance	*Χ*^*2*^	*df*	*p*
*(a) Model comparison table for hits*								
Base	5	4020.9	4043.8	− 2005.5	4010.9			
Intercept	6	4012.7	4040.2	− 2000.3	4000.7	10.2586	1	0.00136**
Linear	7	4014.4	4046.5	− 2000.2	4000.4	0.2601	1	0.61008
Quadratic	8	4012.7	4049.3	− 1998.3	3996.7	3.7507	1	0.05279
*(b) Model comparison table for CRs*								
Base	5	6400.6	6423.5	− 3195.3	6390.6			
Intercept	6	6401.7	6429.2	− 3194.9	6389.7	0.8637	1	0.3527
Linear	7	6403.7	6435.7	− 3194.8	6389.7	0.0347	1	0.8523
Quadratic	8	6404.1	6440.8	− 3194.1	6388.1	1.5453	1	0.2138
*(c) Model comparison table for RTs*								
Base	5	− 2365.6	− 2337.5	1187.8	− 2375.6			
Intercept	10	− 3374.5	− 3318.5	1697.3	− 3394.5	1018.96	5	<.001***
Linear	15	− 3376.2	− 3292	1703.1	− 3406.2	11.6346	5	0.040*
Quadratic	20	− 3372.8	− 3260.6	1706.4	− 3412.8	6.6411	5	0.248

* indicates *p* < .05, ** indicates *p* < .01, *** indicates *p* < .001

**Table 17:** Chosen model coefficients tables for CPT measures in E2 session 2—(a) hits, (b) CRs, and (c) RTs

Fixed effects	Estimate	Std. error	*df*	*t* value	*p*
*(a) Quadratic model coefficients for hits*					
(Intercept)	98.7153	0.5035	60	196.042	< 2e-16***
time^1^	− 0.2615	0.3165	660	− 0.826	0.40899
ISI1	− 0.4167	0.1292	660	− 3.225	0.00132**
time^2^	− 0.6933	0.3165	660	− 2.191	0.02881*
time^1^:ISI1	0.1619	0.3165	660	0.511	6.09E−01
time^2^:ISI1	− 0.6137	0.3165	660	− 1.939	0.05288
*(b) Base model coefficients for CRs*					
(Intercept)	81.215	1.679	60	4.84E + 01	< 2e−16***
time^1^	5.403	1.739	660	3.107	0.00197**
time^2^	− 3.796	1.739	660	− 2.182	0.02943*
*(c) Linear model coefficients for RTs*					
(Intercept)	6.12639	0.0169	59.99	362.691	< 2e−16***
time^1^	0.03903	0.0078	660	4.986	7.87E−07***
ISI1	− 0.08163	0.0031	660	− 25.547	< 2e−16***
time^2^	− 0.00870	0.0078	660	− 1.112	0.267
time^1^:ISI1	− 0.03767	0.0078	660	− 4.813	1.85E−06***

* indicates *p* < .05, ** indicates *p* < .01, *** indicates *p* < .001

*CRs*: None of the models provided a better fit of the data than the base model according to our criterion, and therefore, we chose to use the Base model (Table 16b). Inspection of the model coefficients (Table 17b) and visual inspection of the graph (Fig. 12b) show: 1. a positive linear trajectory modified by a negative quadratic term (i.e., rapidly improving performance in earlier blocks tapering off in later block) during both ISI conditions; and 2. no significant differences in performance between ISIs.

*RTs*: The Linear model provided the best fit of the data according to our criterion (Table 16c),* χ*^*2*^(5) = 11.63*, p* <.05. Inspection of the model coefficients (Table 17c) and visual inspection of the graph (Fig. 12c) show: 1. a positive linear trajectory (i.e., worsening performance) during the 4000 ms ISI condition; 2. a flat trajectory (i.e., constant performance) during the 1000 ms ISI; and 3. significantly faster (i.e., better) performance during the 1000 ms ISI.

#### Session 2 SDT indices

**Fig. 13:**
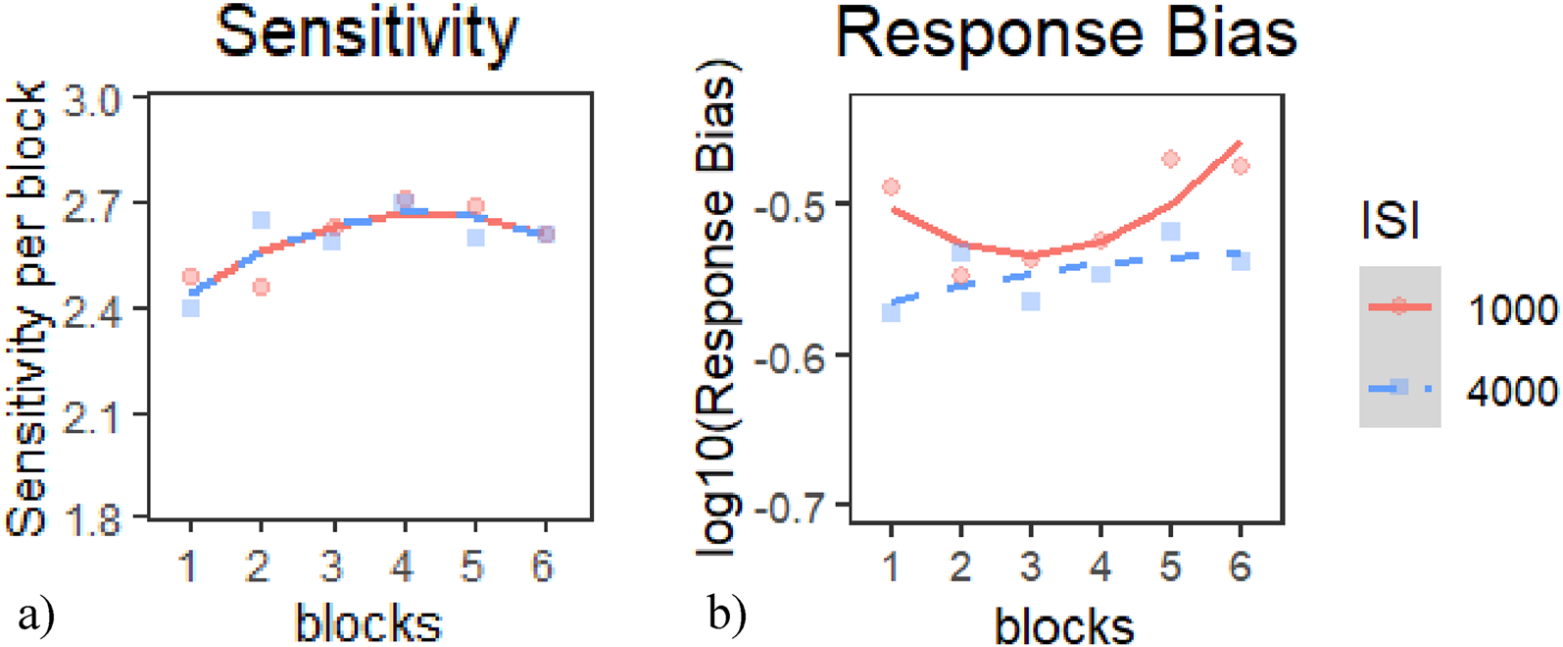
Growth curve analysis graphs for SDT indices in E2 Session 2—**a** sensitivity and **b** response bias. Dots represent observed mean performance in each block and ISI combination, and lines represent the chosen model’s predictions

*Sensitivity*: None of the models provided a better fit of the data than the base model according to our criterion, and therefore, we chose to use the Base model (Table 18a). Inspection of the model coefficients (Table 19a) and visual inspection of the graph (Fig. 13a) show: 1. a positive linear trajectory modified by a negative quadratic term (i.e., rapidly improving performance in earlier blocks tapering off in later block) during both ISI conditions; and 2. no significant differences in performance between ISIs.

**Table 18:** Model comparison tables for SDT indices in E2 session 2—(a) sensitivity and (b) response bias

Model	nPar	AIC	BIC	Loglik	Deviance	*Χ*^*2*^	*df*	*p*
*(a) Model comparison table for sensitivity*								
Base	5	1263.2	1286.1	− 626.59	1253.2			
Intercept	6	1265.1	1292.6	− 626.57	1253.1	0.0496	1	0.8237
Linear	7	1267	1299	− 626.47	1253	0.1925	1	0.6609
Quadratic	8	1268.8	1305.4	− 626.4	1252.8	0.1511	1	0.6975
*(b) Model comparison table for response bias*								
Base	5	− 326.34	− 303.44	168.17	− 336.34			
Intercept	6	− 332.47	− 304.99	172.23	− 344.47	8.1309	1	0.004352**
Linear	7	− 330.56	− 298.5	172.28	− 344.56	0.0927	1	0.760755
Quadratic	8	− 331.49	− 294.86	173.75	− 347.49	2.9313	1	0.086876

* indicates *p* < .05, ** indicates *p* < .01, *** indicates *p* < .001

**Table 19:** Chosen model coefficients tables for SDT indices in E2 session 2—(a) sensitivity and (b) response bias

Fixed effects	Estimate	Std. error	*df*	t value	*p*
*(a) Base model coefficients for sensitivity*					
(Intercept)	2.58775	0.05381	60	48.088	< 2e−16***
Time^1^	0.1415	0.0485	660.0000	2.9150	0.0037**
Time^2^	− 0.1284	0.0485	660.0000	− 2.6470	0.0083**
*(b) Quadratic model coefficients for response bias*					
(Intercept)	− 0.5263	0.0136	60.0000	− 38.7970	< 2e−16***
Time^1^	0.0324	0.0164	660.0000	1.9800	0.0482*
ISI1	0.0191	0.0067	660.0000	2.8670	0.0043**
Time^2^	0.0211	0.0164	660.0000	1.2900	0.1975
Time^1^:ISI1	0.0050	0.0164	660.0000	0.3050	0.7603
Time^2^:ISI1	0.0280	0.0164	660.0000	1.7140	0.0870

* indicates *p* < .05, ** indicates *p* < .01, *** indicates *p* < .001

*Response Bias*: The Quadratic model provided the best fit of the data according to our criterion (Table 18b), *χ*^*2*^(1) = 2.93*, p* <.09. Inspection of the model coefficients (Table 19b) and visual inspection of the graph (Fig. 13b) show: 1. an overall positive linear trajectory (i.e., increasingly conservative) in both ISI conditions; 2. a negative quadratic trajectory (i.e., increasingly liberal then increasingly conservative bias) during the 1000 ms ISI; and 3. response bias was overall liberal, and more liberal during the 4000 ms ISI than in the 1000 ms ISI.

#### Session 2 tracking measure

**Fig. 14:**
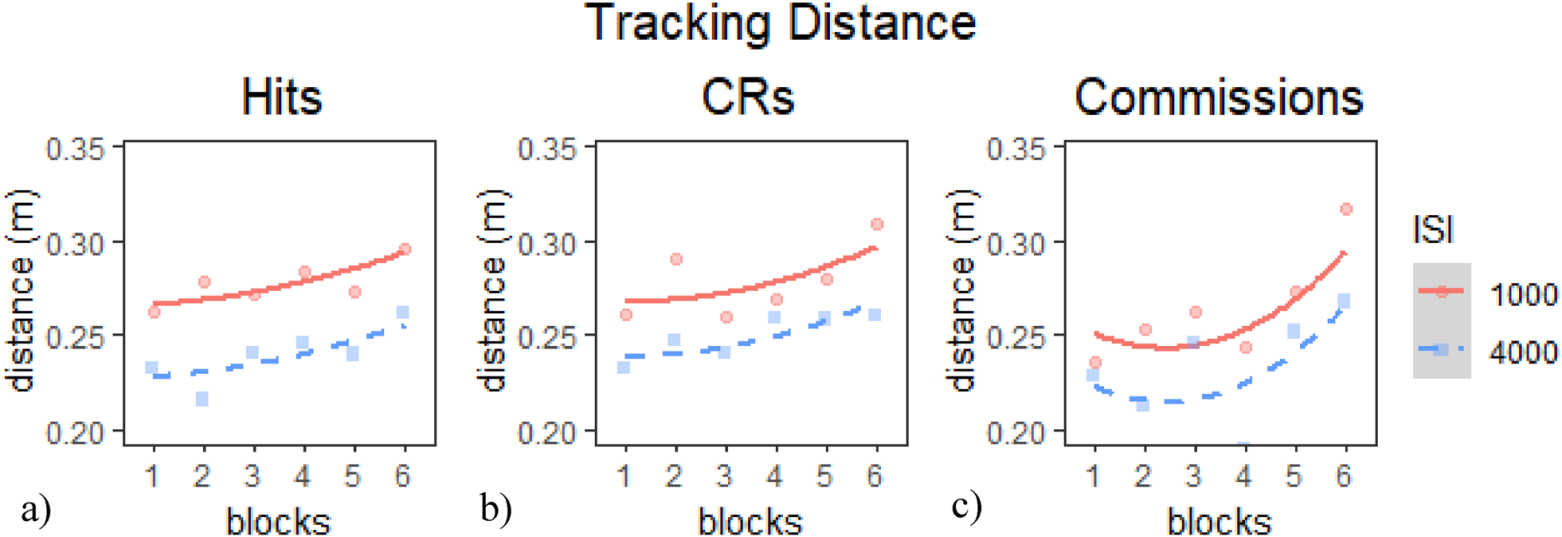
Growth curve analysis graphs for tracking distance measures in E2 session 2—**a** hits, **b** CRs, and **c** commissions. Dots represent observed mean performance in each block and ISI combination, and lines represent the chosen model’s predictions

*Average Tracking Distance during Hits*: The Intercept model provided the best fit of the data according to our criterion (Table 20a), *χ*^*2*^(1) = 44.7*, p* <.001. Inspection of the model coefficients (Table 21a) and visual inspection of the graph (Fig. 14a) show: 1. positive linear trajectories (i.e., worsening performance) during both ISI conditions and 2. highest (i.e., worse) performance during the 1000 ms ISI.

**Table 20:** Model comparison tables for tracking distance measures in E2 session 2—(a) hits, (b) CRs, and (c) Commissions

Model	nPar	AIC	BIC	Loglik	Deviance	*Χ*^*2*^	*df*	*p*
*(a) Model comparison table for tracking distance during hits*								
Base	5	− 1478.8	− 1455.9	744.39	− 1488.8			
Intercept	6	− 1521.5	− 1494	766.75	− 1533.5	44.7072	1	2.29E−11***
Linear	7	− 1519.8	− 1487.7	766.88	− 1533.8	0.2779	1	0.5981
Quadratic	8	− 1518	− 1481.3	766.99	− 1534	0.2106	1	0.6463
*(b) Model comparison table for tracking distance during CRs*								
Base	5	− 1064.6	− 1041.7	537.3	− 1074.6			
Intercept	6	− 1076.5	− 1049	544.26	− 1088.5	13.9211	1	1.91E−04***
Linear	7	− 1074.5	− 1042.5	544.27	− 1088.5	0.0197	1	0.888336
Quadratic	8	− 1074	− 1037.4	545.01	− 1090	1.4852	1	0.222956
*(c) Model comparison table for tracking distance during commissions*								
Base	5	− 386.86	− 365.97	198.43	− 396.86			
Intercept	6	− 389.01	− 363.94	200.5	− 401.01	4.1427	1	4.18E−02*
Linear	7	− 387.22	− 357.98	200.61	− 401.22	0.2157	1	0.64237
Quadratic	8	− 385.23	− 351.8	200.61	− 401.23	0.0068	1	0.93439

* indicates *p* < .05, ** indicates *p* < .01, *** indicates *p* < .001

**Table 21:** Chosen model coefficients tables for tracking distance measures in E2 session 2—(a) hits, (b) CRs, and (c) commissions

Fixed effects	Estimate	Std. error	df	*t* value	*p*
*(a) Intercept model coefficients for tracking distance during hits*					
(Intercept)	2.58E−01	1.02E−02	6.00E + 01	25.354	< 2e−16***
Time^1^	2.29E−02	6.84E−03	6.60E + 02	3.352	0.000847***
Time^2^	4.56E−03	6.84E−03	6.60E + 02	0.666	0.505486
ISI1	1.90E−02	2.79E−03	6.60E + 02	6.801	2.33E−11***
*(b) Intercept model coefficients for tracking distance during CRs*					
(Intercept)	2.64E−01	1.29E−02	6.00E + 01	20.388	< 2e−16***
Time^1^	2.42E−02	9.37E−03	6.60E + 02	2.579	0.010123*
Time^2^	7.01E−03	9.37E−03	6.60E + 02	0.748	0.454634
ISI1	1.44E−02	3.83E−03	6.60E + 02	3.751	1.92E−04***
*(c) Intercept model coefficients for tracking distance during commissions*					
(Intercept)	2.58E−01	1.06E−02	6.00E + 01	24.312	< 2e−16***
Time^1^	2.28E−02	6.13E−03	6.60E + 02	3.724	0.000213***
Time^2^	5.14E−03	6.13E−03	6.60E + 02	0.839	0.402041
ISI1	1.70E−02	2.50E−03	6.60E + 02	6.785	2.59E−11***

* indicates *p* < .05, ** indicates *p* < .01, *** indicates *p* < .001

*Average Tracking Distance during CRs*: The Intercept model provided the best fit of the data according to our criterion (Table 20b), *χ*^*2*^(1) = 13.9*, p* <.001. Inspection of the model coefficients (Table 21b) and visual inspection of the graph (Fig. 14b) show: 1. positive linear trajectories (i.e., worsening performance) during both ISI conditions and 2. highest (i.e., worse) performance during the 1000 ms ISI.

*Average Tracking Distance during Commissions*: The Intercept model provided the best fit of the data according to our criterion (Table 20c), *χ*^*2*^(1) = 4.14*, p* <.05. Inspection of the model coefficients (Table 21c) and visual inspection of the graph (Fig. 14c) show: 1. positive linear trajectories (i.e., worsening performance) during both ISI conditions and 2. highest (i.e., worse) performance during the 1000 ms ISI.

### Discussion

With respect to our questions for both experiments, we can answer positively to both since performance trajectories indeed varied for certain measures and indices across the session time-blocks, as well as between the ISIs. Moreover, the methodological changes we introduced in E2 appear to have achieved their intended goals. In particular, differences between ISI conditions in the dual-task second session were more pronounced, and the tracking distance measure was no longer characterized by the large early practice effects seen in E1 but instead showed a marked vigilance decrement.

In the single-task Session 1, hits improved early but then leveled off later (Fig. 1c—dashed line), showing no differences between ISIs. CRs also showed initial improvement that tapered off, though performance was slightly worse at the 1000 ms ISI (Fig. 1c—solid line). RTs steadily worsened across blocks (Fig. 1b—solid line) but were faster at the 1000 ms ISI. For the SDT indices, sensitivity progressively improved across blocks before tapering off in the final blocks, with no differences between ISIs, while response bias remained stable and liberal across ISIs (Fig. 1a). Overall, these results confirm that the CPT is sensitive to practice changes and, in CRs perhaps to vigilance decrements as well. Although there were baseline differences between the two ISIs in CRs and RTs, the temporal trajectories did not differ between the two ISIs. The overall consistent Session 1 performance patterns in the two experiments further reinforce the paradigm’s reliability. Our main interest, however, lies in the dual-task Session 2.

Compared to E1, Session 2 in E2 produced more pronounced effects across several measures. Hits were again higher in the 4000 ms ISI than in the 1000 ms ISI condition, as in E1. However, in E2, the 1000 ms ISI condition exhibited a curvilinear pattern indicating initial practice gains followed by vigilance decrements (Fig. 1c—solid line), while the 4000 ms ISI condition showed a steady linear vigilance decrement. CRs followed a pattern similar to E1 with early linear practice effects which appeared to taper off toward the later blocks, with no significant differences between ISIs. RTs were slower overall in the 4000 ms ISI, consistent with E1, but in E2, RT showed a progressive linear vigilance decrement in that condition, but RTs in the 1000 ms ISI remained flat across blocks.

Regarding the SDT indices, like in the 1000 ms and 4000 ms conditions in E1, sensitivity improved early and tapered off for both ISIs, again showing no ISI-related differences. In contrast, response bias demonstrated new effects not seen in E1: a curvilinear trajectory during the 1000 ms ISI with performance initially becoming more liberal and subsequently becoming more conservative, and a progressive linear vigilance decrement during the 4000 ms ISI. Overall, response bias remained more liberal in the 4000 ms ISI condition.

Notably, the tracking distance measure, unlike in E1, showed no early practice effect but instead exhibited linear vigilance decrements across the session in all contexts: during hits, CRs, and commissions. Thus, it appears that the additional practice with just the tracking task introduced in E2 was effective in that there were no practice effects in tracking performance, which allowed vigilance decrements to be detected. Notably, while there were baseline differences between the two ISI such that tracking was better in the 4000 ms ISI during all CPT response types, there were no differences in temporal trajectories between the two ISIs. To help the reader understand the overall pattern of results, Appendix C includes a summary table of the findings and their theoretical implications.

Together, these findings suggest a strategic shift in task prioritization between experiments. In E1, participants appeared to prioritize maintaining target detection, whereas in E2, they may have shifted focus toward sustaining CR performance, which remained comparable across ISIs. Accordingly, vigilance decrements in E2 were more apparent in hits and response bias, especially in the 1000 ms ISI, whereas CRs and sensitivity were relatively preserved. Interestingly, RTs mirrored E1 in the 4000 ms ISI (declining steadily), but remained constant in the 1000 ms ISI, suggesting participants prioritized maintaining fast target responses under time pressure. Finally, the consistent linear decrements in tracking distance across hits, CRs, and Commissions further suggest that participants began Session 2 at a stable performance due to the added practice, then gradually declined.

In sum, these results reinforce and extend the interpretation that multiple mechanisms contribute to vigilance decrements in our design. Specifically, the 1000 ms ISI imposed greater demands on executive resources, likely due to greater demands of quick response and task-switching, resulting in performance declines for hits and tracking distance that are consistent with cognitive overload. At the same time, the relative stability of CRs and RTs in this condition suggests that participants may have strategically reallocated effort to preserve certain aspects of performance, consistent with opportunity-cost models. Meanwhile, the consistent linear vigilance decrements observed in the 4000 ms ISI condition for hits and RTs most likely reflect underload, possibly due to the lower demand required in this lower arousal condition.

Overall, the E2 findings not only validate the methodological refinements introduced to address the limitations of E1, but also reveal a more nuanced dissociation between overload- and underload-related performance patterns across task components. These dissociations provide a window into the multiple mechanisms underlying vigilance decrements and set the stage for a broader theoretical integration in the General Discussion.

## General discussion

In this study, we established and validated a methodology to examine sustained attention in the context of a dual-task scenario in which one of the tasks is a discrete go-no-go target-detection task and the other a continuous target-tracking task. Specifically, participants in two experiments performed a demanding CPT (Conners, 2014) for approximately 12 min (single-task session) and then performed the CPT simultaneously with a driving-based tracking task (Mahr et al., 2012; Math et al., 2013) for the same duration (dual-task session). We utilized GCAs in combination with a mixed-effects modeling framework to analyze and characterize temporal performance trajectories using polynomial functions across three CPT measures (hits, CRs, and RTs), two SDT indices (sensitivity and response bias), and one tracking measure (tracking distance).

Our first question for this study was whether performance would change across the six blocks for each of the measures and indices associated with the CPT and tracking tasks. Our second question for this study was whether the temporal trajectories of any of our measures would differ when target presentation rates are faster compared to when they are slower. Across the two experiments, we were able to answer positively to both questions since performance trajectories indeed varied for certain measures and indices across the session blocks, as well as between the ISI conditions. This applies not only to the dual-task Session 2, which is the primary interest in this study, but also to the single-task Session 1, which shows that our CPT was sensitive enough to detect both practice and vigilance decrements.

Taken together, the results from Session 2 of both experiments demonstrate that operators are indeed susceptible to vigilance decrements during complex multitasking scenarios (Fisk & Schneider, 1981; Gartenberg et al., 2018; Thomson et al., 2015). However, the variation in performance trajectories that we observed across the measures, indices, and experiments suggests that the onset, duration, and magnitude of these decrements may be shaped by multiple dimensions of *task demand* (Hancock et al., 1995; Salvucci & Taatgen, 2008; Wickens, 2008). Indeed, we argue here that task demand is not a singular construct but is instead composed of several components with each playing a distinct role in how participants allocate attention between the target detection and tracking tasks within our paradigm.

One component is *task pacing*, or the systematic manipulation of target presentation rates (i.e., interstimulus intervals, or ISIs). In both experiments, faster target presentation rates result in greater demand due to increased time pressure and increased pressure on executive processes necessary for coordinating the two tasks. This was evident by declines in hits and tracking performance seen in the fastest ISI in both experiments. However, performance was not uniformly worse at faster ISIs, nor uniformly better at slower ISIs in all measures, as illustrated by the similarly stable CRs and sensitivity in the 1000 ms and 4000 ms ISI. Moreover, in E2, RTs showed a greater vigilance decrement in the 4000 ms condition, suggesting that RTs may be more affected by waning arousal than by increases in task demand. This heterogeneity among the different measures indicates that the effects of task pacing depend on the specific cognitive processes underlying each measure (Humphrey et al., 2024; Langner & Eickhoff, 2013; Matthews et al., 2017). This in turn reinforces the utility of using the CPT, which generates multiple measures of performance.

Another critical component is the requirement for *target detection accuracy*, measured in the CPT as the ability to detect and respond to true targets, while inhibiting responses for non-targets. These processes were measured by hits and CRs, respectively, as well as the SDT sensitivity index. In E1, hits remained relatively flat but were highest in the 4000 ms ISI, while CRs and sensitivity followed curvilinear patterns, showing the greatest decrements in the 1000 ms and 4000 ms ISIs. In E2, hits again were lowest in the 4000 ms ISI but curvilinear in the 1000 ms ISI, while CRs and sensitivity were also curvilinear, but did not differ between ISIs and showed pronounced practice effects. These findings suggest that although faster pacing increases overall task difficulty, its impact on target detection accuracy varies by process. Specifically, measures associated with executive control (CRs and sensitivity) appear more broadly susceptible to vigilance decrements across pacing conditions, whereas measures reflecting more automated responding (hits) are sensitive to faster pacing and may also be influenced by arousal (Luna et al., 2018, 2021a, 2021b, 2022a, 2022b; Martínez-Pérez et al., 2022; Matthews & Davies, 1998).

A third critical component is the *processing speed* requirement, measured in the CPT by response times (i.e., RTs) to targets, and in the tracking task as the distance between the target and the participant-controlled indicator. In E1, RTs showed consistent linear vigilance decrements but were slowest in the 4000 ms ISI, while tracking followed curvilinear patterns but was overall worse in the 1000 ms ISI. In E2, RTs only showed a linear vigilance decrement in the 4000 ms ISI, which was slower than the flat performance in the 1000 ms ISI, while tracking followed consistent linear vigilance decrements and performance were again worse overall in the 1000 ms ISI. These findings suggest that while both RTs and tracking reflect relatively automated motor processes (Fisk & Schneider, 1981; Körber et al., 2015; Meuter et al., 2005), the vigilance decrements observed in these two measures may be influenced by different underlying mechanisms.

Finally, a fourth critical component of task performance in our paradigm is *task-switching*, the requirement to continually allocate attention between the CPT and tracking tasks. While not directly measured, task-switching demands were inferred through ‘cross-task interactions,’ where tracking performance was examined in relation to specific events in the CPT (Egner, 2023; Koch et al., 2018; Meyer & Kieras, 1997; Monsell, 2003). These effects were most apparent in the 1000 ms ISI condition of E2, which showed curvilinear patterns in both hits and response bias, and consistent linear declines in tracking performance. Compared to E1, these patterns suggest that additional practice improved participants’ ability to manage switching demands by reallocating attention toward the more cognitively demanding CPT. These findings highlight task-switching as a key driver of dual-task difficulty and as a moment-to-moment regulator of attentional control (Mitchell, 2018; Pashler, 1994; Poljac et al., 2018; Wickens, 2002). Ultimately, even modest changes in dual-task parameters, such as prior practice or task pacing, can significantly influence how attention and effort are coordinated between tasks (Ruthruff et al., 2001; Strobach & Torsten, 2017).

### Theoretical implications

The findings from our study support a multi-mechanism account of vigilance decrements that incorporates elements of cognitive overload, cognitive underload, and opportunity-cost models. Each model helps explain different aspects of performance decline under specific task demands, reinforcing the idea that vigilance decrements are not driven by a single, uniform cause but rather emerge from how participants allocate limited attentional resources in response to changing cognitive demands across tasks, which vary dynamically based on task pacing, as well as requirements for target detection, processing, and task coordination.

The cognitive overload, or resource depletion, account (Caggiano & Parasuraman, 2004; Fisk & Scerbo, 1987; Gartenberg et al., 2018; Helton & Warm, 2008; Matthews et al., 1993; Wiener et al., 1984) was most evident in the late-session linear declines in CRs and sensitivity, which emerged in both the faster (1000 ms) and slower (4000 ms) ISI conditions across experiments. Similarly, the tracking measures also showed consistent temporal decline (in later sessions in E1 and from the very start in E2). These vigilance decrements were likely due to the cumulative effort required to maintain target detection accuracy under increasing dual-task demands. The fact that these declines occurred across ISI conditions suggest that the task switching demands, rather than task pacing alone, contribute to resource depletion.

In contrast, the cognitive underload account (Cummings et al., 2016; Greenlee et al., 2018; McBain, 1970; Scerbo et al., 1992) is most evident in the uniform gradual declines in RTs in the 4000 ms ISI in E2. These patterns suggest that slower task pacing may have led to lower levels of arousal and task engagement, thereby impairing sustained responsiveness to stimuli over time. Thus, vigilance decrements observed under slower pacing appear to stem not from the burden of multitasking, but rather from conditions that insufficiently sustained task engagement and arousal.

Lastly, the opportunity-cost account (Kurzban et al., 2013) is most evident in two aspects of the results. The first is the curvilinear trajectories of hits and response bias in the 1000 ms ISI condition. In the 1000 ms ISI condition, participants appeared to strategically reallocate cognitive resources over time, maintaining performance on certain task elements (e.g., fast responses), while allowing declines in others (e.g., accuracy, or conservativeness in responding). This pattern suggests that participants were continuously evaluating the relative utility of sustaining effort across competing task demands, particularly under faster task pacing. The second aspect of our result that supports the opportunity-cost account is the difference between the two experiments in for which task requirement participants showed similar temporal trajectories across ISIs (hits in E1 vs. CRs in E2). These findings reinforce the idea that vigilance decrements can arise not only from excessive or insufficient task demand, but also from motivational factors that shape how attentional resources are distributed across tasks.

As mentioned earlier, Appendices B and C provide summary tables of the results of both experiments and their theoretical implications. The reader is encouraged to consider both tables for the complete picture of how our results show that different mechanisms may operate in parallel or interactively under varying task demands.

Importantly, our findings also highlight conditions under which performance can be protected from vigilance decrements (Akre, 2017; Arrabito et al., 2007; Hancock et al., 2016; Murata et al., 2025). First, vigilance decrements linked to cognitive overload may be mitigated by reducing the frequency of task-switching (Ross et al., 2014), optimizing task pacing to minimize excessive time pressure (Murray & Amaya, 2024), and providing sufficient task-specific practice to support automation of component skills (Parasuraman & Giambra, 1991). Second, decrements linked to cognitive underload may be mitigated by introducing moderate, varied demands to sustain engagement during low-arousal periods, or by incorporating brief rest intervals that refresh attentional resources (Ariga & Lleras, 2011; Atchley et al., 2014; Ross et al., 2014). Notably, the absence of pronounced vigilance decrements in the 2000 ms ISI condition in E1 suggests there may be a ‘sweet spot’ for specific dual-task scenarios where cognitive load is sufficiently engaging but not overwhelming, providing relative protection from both underload and overload effects (McWilliams & Ward, 2021; Wiener et al., 1984; Yerkes & Dodson, 1908; Young et al., 2015). Finally, decrements linked to opportunity costs may be mitigated by increasing the perceived utility of task elements, for example, by introducing reward structures or clearly defined performance goals, thereby sustaining motivation to allocate resources effectively across tasks (Esterman et al., 2014; Gutzwiller et al., 2015).

### Practical applications

Aside from clarifying some of the mechanisms involved with sustaining attention, our study highlights the importance of using ecologically valid methods to examine multitasking performance in controlled laboratory settings. Our CPT paradigm, which requires participants to respond to targets while withholding responses to non-targets, mirrors real-world scenarios such as military targeting operations that demand rapid and accurate decision making under pressure (Munnik et al., 2020; Wilson, 2015). Moreover, the combined CPT and driving-based tracking task simulates the demands of drone operations, which require precision across both continuous and discrete control tasks, such as managing drone swarms and navigating dynamic environments (Aguilar et al., 2017; Aswini et al., 2018; Chérif et al., 2018; Yao et al., 2015; Zieliński, 2021). Synthesizing these insights, our results emphasize that human–machine interface design must consider and empirically validate subtle task parameters to support optimal performance across dimensions such as practice, executive resources, engagement, and arousal.

This work also contributes to ongoing efforts to improve operator vigilance in military contexts. The United States Department of Defense has increasingly invested in real-time measures of cognitive load, effort allocation, and task disengagement through physiological monitoring tools like eye tracking and EEG (Kim, 2016; Nelson et al., 2019; Weightman et al., 2017; Zhang et al., 2024). While automation may improve efficiency, it can also reduce arousal and alter task demands in ways that undermine performance (McWilliams & Ward, 2001). This has implications not only for reducing operational errors during critical missions (Abich IV et al., 2017; Nicolae et al., 2016; Shappell & Wiegmann, 2004; Thomas & Russo, 2007), but also for developing cognitive rehabilitation strategies to support recovery and performance in service members with brain injuries (Zotey et al., 2023).

Similar challenges are present in aviation, where both pilots and air traffic controllers must sustain attention over extended periods while managing complex multitasking demands (Balta et al., 2024; Bongo & Seva, 2022; Casner & Schooler, 2015; Hitchcock et al., 2003; McGee et al., 1997, 1998; Metzger & Parasuraman, 2017; Sallinen et al., 2017; Stearman & Durso, 2016; Terenzi et al., 2024). Fatigue, high workload, and reduced arousal can lead to attentional failures with potentially catastrophic consequences, as seen in the January 2025 mid-air collision at Ronald Reagan Washington National Airport, where human error linked to vigilance decrement was identified as a contributing factor (Baldor et al., 2025). Addressing these risks requires investment in targeted training, communications protocols, and supportive technologies to help operators sustain attention and prevent errors in high stake environments (Levine, 2009).

### Limitations and future research

This study has several limitations that should be addressed in future work. First, unlike other studies that implemented similar paradigms (e.g., Buckley et al., 2013), our paradigm utilized fixed-order sessions and did not include a tracking-only session. We did this because we wanted to give participants more time to practice the CPT (compared to the tracking task) so as to focus on the changes in performance that occur during the more demanding dual-task session. While our study was successful in advancing the understanding of the origins of vigilance decrements during dual-task performance, we could not derive any conclusions about the differences between dual-task and single-task performance for either task. Thus, although using these fixed-order sessions was necessary for the purposes of this study, it may still be useful for future studies to compare vigilance decrements in single- and dual-task contexts by manipulating the order of single- and dual-task blocks.

Second, we did not include self-report data from validated questionnaires about mind-wandering since we believed they would be minimally informative given the design of our study. However, in line with other related vigilance studies (Körber et al., 2015; Mooneyham & Schooler, 2013; Seli et al., 2016; Smallwood et al., 2004; Thomson et al., 2015; Weinstein, 2018; Yanko & Spalek, 2014), it may be worthwhile to also assess whether and to what extent operators engage in mind-wandering activities during demanding multitasking scenarios. This could provide useful insight for assessing more recent theories for vigilance decrements not directly examined in this study, such as the resource-control account (Thomson et al., 2015). That said, it may also be interesting to collect continuous physiological measures of workload and stress, such as heart rates, eye movement activity, and EEG (Hancock, 1989; Mehrabi & Kim, 2022), to further examine the roles of both overload and underload on sustained multitasking performance.

Third, while the duration of sustained attention tasks can range from several minutes to hours, our paradigm specifically measured performance within a relatively short time window (approximately 12-min). This was not a limitation per se, since our interest was on the performance changes that much literature has shown to occur during this relatively short period (e.g., Conners et al., 2003; Loh et al., 2004; Roach et al., 2006). Nevertheless, extending the duration of our paradigm would be a useful contribution to the literature since this relates more to the scenarios operators typically encounter in the real world (Canisius & Penzel, 2007; Mackie, 1987; Mackworth, 1968; Popp et al., 2015).

## Conclusion

In this paper, we examined the time course of vigilance decrements that occur when operators perform demanding multitasking activities. Our findings suggest that cognitive overload, underload, and opportunity costs play significant roles in vigilance performance and that dissociating the underlying mechanisms involved in task performance can help better interpret measures from common CPTs used in sustained attention research. In addition to contributing to the theoretical understanding of attention and multitasking performance, the insights gained from our study can inform the development of strategies and interventions to mitigate vigilance decrements and improve performance in demanding work environments, both civilian and military.

## Data Availability

The datasets used and analyzed during the current study are available from the corresponding author on reasonable request.

## Appendix A

See Table

**Table 22:** Growth curve models for fitting CPT, SDT, tracking measures for participant *i* at block *j* for E1 and E2

Model	Equation
1. Base	*Ƴ*_*ij*_ = *β*_0*i*_ + *β*_*1*_ * Block_*j*_ + *β*_*2*_ * Block_*j*_^2^ + *ε*_*i*_
	*β*_0i_ = *β*_0_ + ζ_0i_ *β*_0_ = ζ_1_ *β*_1_ = ζ_2_ *β*_2_ = ζ_3_
2. Intercept	*Ƴ*_*ij*_ = *β*_0*i*_ + *β*_*1*_ * Block_*j*_ + *β*_*2*_ * Block_*j*_^2^ + *ε*_*i*_
	*β*_0i_ = *β*_0_ + ζ_0i_ *β*_0_ = ζ_1_* ISI *β*_1_ = ζ_2_ *β*_2_ = ζ_3_
3. Linear	*Ƴ*_*ij*_ = *β*_0*i*_ + *β*_*1*_ * Block_*j*_ + *β*_*2*_ * Block_*j*_^2^ + *ε*_*i*_
	*β*_0i_ = *β*_0_ + ζ_0i_ *β*_0_ = ζ_1_* ISI *β*_1_ = ζ_2_* ISI *β*_2_ = ζ_3_
4. Quadratic	*Ƴ*_*ij*_ = *β*_0*i*_ + *β*_*1*_ * Block_*j*_ + *β*_*2*_ * Block_*j*_^2^ + *ε*_*i*_
	*β*_0i_ = *β*_0_ + ζ_0i_ *β*_0_ = ζ_1_* ISI *β*_1_ = ζ_2_* ISI *β*_2_ = ζ_3_* ISI

22.

## Appendix B

See Table

**Table 23:** Interpretation table for session 2 measures in experiment 1

Measure	ISI	Performance summary	Temporal pattern	Key process	Theoretical implication(s)
Hits	1000	Lowest Accuracy	Flat	Target Detection (automated)	Underload + Opportunity Costs
	2000	Moderate Accuracy	Flat		
	4000	Highest Accuracy	Flat		
CRs	1000	Lowest Accuracy	Curvilinear Decline	Target Detection (executive)	Overload
	2000	Stable	Flat		
	4000	Lowest Accuracy	Curvilinear Decline		
RTs	1000	Fastest Responses	Linear Decrement	Processing Speed	Underload
	2000	Intermediate RTs	Linear Decrement		
	4000	Slowest Responses	Linear Decrement		
Sensitivity	1000	Lowest Overall	Curvilinear Decline	Detection + Discrimination	Overload + Opportunity Costs
	2000	Stable	Flat		
	4000	Lowest Overall	Curvilinear Decline		
Response bias	1000	Most Conservative	Flat (Liberal)	Task-Switching + Decision Threshold	Opportunity Costs
	2000	Moderate Bias	Flat (Liberal)		
	4000	Most Liberal	Flat (Liberal)		
Tracking (Hits)	1000	Worst Tracking	Linear Decrement	Task-Switching	Overload + Opportunity Costs
	2000	Moderate	Curvilinear		
	4000	Moderate	Curvilinear		
Tracking (CRs)	1000	Worst Tracking	Curvilinear	Task-Switching	Overload + Opportunity Costs
	2000	Moderate	Curvilinear		
	4000	Best Tracking	Curvilinear		
Tracking (Comm.)	1000	Worst Tracking	Flat	Task-Switching	Overload + Opportunity Costs
	2000	Moderate	Flat		
	4000	Best Tracking	Flat		

23.

## Appendix C

See Table

**Table 24:** Interpretation table for session 2 measures in experiment 2

Measure	ISI	Performance summary	Temporal pattern	Key process	Theoretical implication(s)
Hits	1000	Lowest Accuracy	Curvilinear	Target Detection (automated)	Underload + Opportunity Costs
	4000	Highest Accuracy	Flat		
CRs	1000	No Difference	Curvilinear	Target Detection (executive)	Overload
	4000	No Difference	Curvilinear		
RTs	1000	Fastest Responses	Flat	Processing Speed	Underload
	4000	Slowest Responses	Linear Decrement		
Sensitivity	1000	No Difference	Curvilinear	Detection + Discrimination	Overload + Opportunity Costs
	4000	No Difference	Curvilinear		
Response bias	1000	Most Liberal	Curvilinear	Task-Switching + Decision Threshold	Opportunity Costs
	4000	Most Conservative	Linear		
Tracking (hits)	1000	Worst Tracking	Linear Decrement	Task-Switching	Overload + Opportunity Costs
	4000	Best Tracking	Linear Decrement		
Tracking (CRs)	1000	Worst Tracking	Linear Decrement	Task-Switching	Overload + Opportunity Costs
	4000	Best Tracking	Linear Decrement		
Tracking (Comm.)	1000	Worst Tracking	Linear Decrement	Task-Switching	Overload + Opportunity Costs
	4000	Best Tracking	Linear Decrement		

24.
